# The distribution of runs of homozygosity in the genome of river and swamp buffaloes reveals a history of adaptation, migration and crossbred events

**DOI:** 10.1186/s12711-021-00616-3

**Published:** 2021-02-27

**Authors:** Nicolo P. P. Macciotta, Licia Colli, Alberto Cesarani, Paolo Ajmone-Marsan, Wai Y. Low, Rick Tearle, John L. Williams

**Affiliations:** 1 Dipartimento di Agraria, Università degli Studi di Sassari, Sassari, Italia; 2 Dipartimento di Scienze Animali, della Nutrizione e degli Alimenti-DIANA, Università Cattolica del Sacro Cuore, Piacenza, Italia; 3 Centro di Ricerca sulla Biodiversità e sul DNA Antico-BioDNA, Università Cattolica del Sacro Cuore, Piacenza, Italia; 4 Department of Animal and Dairy Science, University of Georgia, Athens, GA USA; 5 Centro di Ricerca Nutrigenomica e Proteomica-PRONUTRIGEN, Università Cattolica del Sacro Cuore, Piacenza, Italia; 6 The Davies Research Centre, School of Animal and Veterinary Sciences, University of Adelaide, Roseworthy, SA 5371 Australia

## Abstract

**Background:**

Water buffalo is one of the most important livestock species in the world. Two types of water buffalo exist: river buffalo (*Bubalus bubalis bubalis*) and swamp buffalo (*Bubalus bubalis carabanensis*). The buffalo genome has been recently sequenced, and thus a new 90 K single nucleotide polymorphism (SNP) bead chip has been developed. In this study, we investigated the genomic population structure and the level of inbreeding of 185 river and 153 swamp buffaloes using runs of homozygosity (ROH). Analyses were carried out jointly and separately for the two buffalo types.

**Results:**

The SNP bead chip detected in swamp about one-third of the SNPs identified in the river type. In total, 18,116 ROH were detected in the combined data set (17,784 SNPs), and 16,251 of these were unique. ROH were present in both buffalo types mostly detected (~ 59%) in swamp buffalo. The number of ROH per animal was larger and genomic inbreeding was higher in swamp than river buffalo. In the separated datasets (46,891 and 17,690 SNPs for river and swamp type, respectively), 19,760 and 10,581 ROH were found in river and swamp, respectively. The genes that map to the ROH islands are associated with the adaptation to the environment, fitness traits and reproduction.

**Conclusions:**

Analysis of ROH features in the genome of the two water buffalo types allowed their genomic characterization and highlighted differences between buffalo types and between breeds. A large ROH island on chromosome 2 was shared between river and swamp buffaloes and contained genes that are involved in environmental adaptation and reproduction.

**Supplementary Information:**

The online version contains supplementary material available at 10.1186/s12711-021-00616-3.

## Background

The domestic water buffalo represents a fundamental livestock resource for rural populations in many areas of the world, providing milk, meat and traction power. This species is also farmed in intensive dairy systems [1, 2] and is most famous for the production of mozzarella cheese, which has a high market value. In the last 50 years, the world buffalo stock has shown a huge increase, from 88,321,807 in 1961 to 200,967,747 heads in 2017 [3], although there are large regional variations. The largest increases in number of buffaloes have occurred in India, Pakistan, and China, whereas the largest relative increase is found in Italy and Brazil (> 200%).

Two types of water buffalo exist: the river buffalo (*Bubalus bubalis bubalis*) and the swamp buffalo (*Bubalus bubalis carabanensis*). However, it is still under discussion if they should be considered as two distinct species, subspecies, or ecotypes. The two buffalo types have a different number of chromosomes due to a tandem fusion between chromosomes 4 and 9 in the swamp buffalo, which results in 50 and 48 chromosomes in the river and swamp buffalo, respectively [4]. They can interbreed and their crosses (2n = 49) are generally fertile [5], although some authors have suggested that the occurrence of unbalanced gametes may reduce fertility [6]. A number of ancient and recent events of admixture between the two types has been recorded [7]. River buffaloes are farmed from India and Pakistan to South-East and South Europe, North Africa and South America, whereas swamp buffaloes are mostly found in East and South East Asia.

Two domestication events occurred in water buffalo about 7000 years ago, on the Indian sub-continent for river buffalo and eastern Asia for swamp buffalo [8, 9]. River and swamp buffaloes exhibit ecological and behavioral differences and are characterized by distinct biodiversity patterns. The river type consists of breeds that show distinct phenotypes as a result of selection, whereas the swamp type is represented mostly by local and unselected populations that are adapted to specific environments [7].

The water buffalo genome was recently sequenced by an International Consortium [10] and a higher quality genome assembly was released in February 2019 [11] with associated annotations (https://www.ncbi.nlm.nih.gov/genome/annotation_euk/Bubalus_bubalis/101/).

During the sequencing project, a medium-density 90 K SNP bead chip was developed in collaboration with Affymetrix (Axiom) using single nucleotide polymorphism (SNP) positions that were based on the *Bos taurus* reference sequence [12]. This 90 K chip has facilitated studies for the detection of candidate genes for milk production [2, 13–15], characterization of breeds, identification of signatures of selection [16], and investigation of the genetic histories of river and swamp populations [7]. Most of SNPs on the buffalo 90 K Axiom SNP chip are now annotated based on the high-quality buffalo genome sequence.

Classical population genetic approaches that are used to study biodiversity, demography, population structure, and inbreeding, can be extended by investigating the distribution of runs of homozygosity (ROH). ROH are continuous stretches of homozygous genotypes that are found along the genome [17]. The proportion of the genome that carries ROH is an indicator of the actual inbreeding level of individuals and populations, whereas pedigree information estimates the expected level of inbreeding [18, 19]. Moreover, the length of ROH is an indicator of the history of inbreeding events: long ROH are evidence of recent inbreeding and short ROH are indicators of ancient inbreeding [18, 19]. ROH can also result from artificial or natural selection, i.e. homozygous genotypes arise from the fixation of favorable alleles at selected loci and from the action of the genomic hitchhiking in surrounding regions. A ROH-based genomic relationship matrix implemented in a genomic best linear unbiased prediction (GBLUP) model allows the prediction of breeding values [20]. ROH are also used to detect selective sweeps in which alleles have become fixed [21, 22], and to map recessive alleles [23]. Recently, ROH distribution was investigated in river buffalo to study their genomic inbreeding and scan the genome for ROH islands [24, 25].

Here, we report the distribution and features of ROH in river and swamp buffalo populations, and use the information for an in-depth genomic investigation of domestic water buffalo to elucidate the genomic structure of different breeds and populations.

## Methods

### Data

The dataset consisted of genotypes for 185 river and 153 swamp buffaloes from 15 river and 13 swamp breeds or populations (Table 1). Animals were sampled during the buffalo genome sequencing project by the partners of the International Buffalo Consortium [12] between years 2011 and 2012, before Directive 2010/63/EU came into force (i.e., 1 January 2013). All experimental procedures complied with the former EU Directive 86/609/EEC, according to which approval from dedicated animal welfare/ethics committee was not necessary. The permission to carry out the sampling at each farm was obtained directly from the owners. All the samples were collected during routine veterinary checks and in accordance with local/national regulations and ethical rules in force at the time of sampling.

**Table 1:** Composition of the animal sample used in the study

Breed/population	Country	Symbol	Number of animals
	River type		
Murrah	Brazil	RIVBR_MUR	30
Colombian	Colombia	RIVCO	12
Egyptian	Egypt	RIVEG	15
Azari	Azerbaijan	RIVIR_AZA	9
Khuzestani	Iran	RIVIR_KHU	10
Mazandarani	Iran	RIVIR_MAZ	8
Mediterranean	Italy	RIVIT_MED	30
Mediterranean	Mozambique	RIVMZ	7
Murrah	Bulgaria	RIVPH_BU	8
Murrah	India (Philippines)	RIVPH_IN	4
Aza Kheli	Pakistan	RIVPK_AZK	3
Kundhi	Pakistan	RIVPK_KUN	10
Nili-Ravi	Pakistan	RIVPK_NIL	15
Mediterranean	Romania	RIVRO	9
Anatolian	Turkey	RIVTR_ANA	15
	Swamp type		
Enshi	China	SWACN_ENS	15
Fuling	China	SWACN_FUL	15
Guizhou	China	SWACN_GUI	11
Hunan	China	SWACN_HUN	14
Yangzou	China	SWACN_YAN	12
Yibin	China	SWACN_YIB	15
Java	Indonesia	SWAIN_JAV	12
Nusa tenggara	Indonesia	SWAIN_NUT	7
Sumatra	Indonesia	SWAIN_SUM	8
South Sulawesi	Indonesia	SWAIN_SUW	10
	Philippines	SWAPH	21
	Thailand	SWATH_THS	5
	Thailand	SWATH_THT	8

All the animals were genotyped with the 90 K Affymetrix Axiom^®^ Buffalo Genotyping Array at the Affymetrix laboratory (Santa Clara, CA, USA). The array was originally developed based on the *Bos taurus* sequence [12]. In the present work, we remapped the markers to the latest version of the buffalo genome assembly [11]. Since the SNP array was developed mainly from sequence data of river type buffaloes, it is moderately affected by ascertainment bias (AB) when it is used to genotype swamp buffalo individuals, as described in a previous study [7]. For this reason, we built and analyzed three datasets: (i) a complete dataset (ALL_DATA) that included all river and swamp animals and was used to compare the two water buffalo types, and (ii) two type-specific datasets that contain only polymorphic SNPs and animals from either buffalo type (RIVER_DATA and SWAMP_DATA) and that were used to analyze within-type differences between breeds or populations.

Initially, all SNPs and animals were quality-controlled as a single set. An animal was discarded if its individual call rate was lower than 0.95 and a SNP was discarded if the call rate was lower than 0.95, and the minor allele frequency (MAF) was lower than 0.01. Additional filtering steps were carried out separately for each of the three datasets. For the ALL_DATA set, only SNPs that were polymorphic in the swamp buffaloes were retained, according to the approach followed by [7] to reduce the impact of ascertainment bias. In the RIVER_DATA and SWAMP_DATA sets, SNPs that deviated statistically from Hardy Weinberg equilibrium expectations (P < 0.00001) were also discarded. After quality control, 17,784 SNPs were retained in the ALL_DATA set, and 46,891 and 17,690 SNPs were retained in the RIVER_DATA and SWAMP_DATA sets, respectively. MAF statistics and linkage disequilibrium (LD) plots for the two types are in (see Additional file 1: Figure S1, Additional file 2 Table S1).

### Detection of runs of homozygosity

Defining the settings for the detection of ROH is a crucial point because of their effect on the results. The detection of ROH was carried out separately for each of the three datasets using the Zanardi pipeline [26]. In the present work, we adopted criteria that were previously used on a medium-density cattle SNP panel [27]: (i) minimum number of SNPs included in a ROH = 15; (ii) minimum ROH length = 1 Mb; (iii) maximum distance between two consecutive SNPs in a ROH = 1 Mb; and (iv) no heterozygous or missing genotypes were allowed. Sliding windows were not used for the detection of ROH. A recent paper on Iranian river buffalo ROH [24] set a more stringent criterion for the minimum number of SNPs included in a ROH but allowed for the presence of one heterozygous or one missing genotype in a ROH. Given the huge reduction in SNP density in the ALL_DATA set, a less stringent threshold for the minimum number of SNPs was adopted in this study but no heterozygous or missing genotypes were allowed.

The following statistics were calculated: number of ROH per animal (n_ROH), average ROH length (l_ROH), ROH distribution across five classes of length that are usually considered for cattle and sheep (1 < Mb ≤ 2; 2 < Mb ≤ 4; 4 < Mb ≤ 8; 8 < Mb ≤ 16; and Mb > 16). The total number of ROH could include exactly the same chromosomal segment repeated in different animals. Thus, a unique ROH was defined as a homozygous segment that starts and finishes at exactly the same precise chromosomal positions [28]. We also checked the distribution of unique ROH among breeds and animals.

The ROH count per SNP (SNP_ROH_), i.e., the number of animals that have a given SNP in a ROH was also calculated [23]. A SNP that has an SNP_ROH_ value in the top 1% of the distribution was considered as significant [28, 29]. Thus, based on SNP_ROH_, genomic regions that contained uninterrupted sequences of significant SNPs were defined as ROH islands.

The ROH-based inbreeding coefficient (F_ROH_) for each animal was calculated as the ratio between the length of the genome covered by ROH and the total genome length [30]. Finally, a ROH based-genomic relationship matrix was calculated (**G**_**ROH**_) by coding SNP genotypes as follows: 0 = heterozygous; 1 = homozygous not included in a ROH; and 2 = homozygous included in a ROH. SNP coding was based on their presence/absence in ROH longer than 4 Mb. **G**_**ROH**_ was then calculated according to [31] for all three datasets.

The functions of the genes included in ROH islands were investigated, with particular attention to regions where significant markers according to their SNP_ROH_ were located. Annotated genes were retrieved from the National Centre for Biotechnology Information (NCBI) databases.

In order to compare the results of ROH detection, particularly their chromosomal location and the length of ROH islands, with more widely used parameters, we calculated the Wright fixation index (F_ST_) for the ALL_DATA set using the equation proposed by [32]. River and swamp buffaloes were compared. F_ST_ interpretation was improved by removing noise from the raw signals with a locally weighed scatterplot smoothing (LOWESS) procedure [33]. A threshold based on F_ST_ distribution was adopted [33–35], and SNPs that were three standard deviations from the mean were considered relevant.

### Statistical analysis

The effects of buffalo type, breed and chromosome on ROH length and n_ROH were tested. Because of the markedly skewed distribution of these two variables, a generalized mixed linear model with a lognormal distribution was used to perform a statistical analysis. In particular, ROH length in the ALL_DATA set was analyzed with the following model:

$$\mathrm{y}=\mu +\mathrm{TYPE}+\mathrm{CHROM}+\mathrm{BREED}\left(\mathrm{TYPE}\right)+\mathrm{animal}+\mathrm{e},$$

where $\mathrm{y}$ is the ROH length, $\mu$ is the overall mean, $\mathrm{TYPE}$ is the fixed effect of buffalo type (river vs swamp), $\mathrm{CHROM}$ is the fixed effect of the chromosome (24 levels), $\mathrm{BREED}(\mathrm{TYPE})$ is the fixed effect of the breed nested within type, $\mathrm{animal}$ is the random effect of the animal (338 levels), and $\mathrm{e}$ is the random residual. The two random effects were assumed to be normally distributed with parameters ($\mathbf{0},\mathbf{I}{\sigma }_{a}^{2}$) and (${\mathbf{0},\mathbf{I}\sigma }_{e}^{2}$), where $\mathbf{I}$ is an identity matrix and ${\sigma }_{a}^{2}$ and ${\sigma }_{e}^{2}$ are variance components associated with the animal and residual random effects, respectively. The repeatability, i.e., the contribution of the animal variance to the total variance was calculated as: ${\mathrm{r}}_{\mathrm{p}}={\sigma }_{a}^{2}/{(\sigma }_{a}^{2}+{\sigma }_{e}^{2})$. This parameter expresses the average correlation between l_ROH values within animals.

The number of ROH per animal (one measure per individual) was analyzed with a generalized linear model that included the fixed effects of type and breed nested within type.

In the RIVER_DATA and SWAMP_DATA sets, ROH length was analyzed with the following generalized mixed linear model:

$$\mathrm{y} =\mu +\mathrm{BREED}+\mathrm{CHROM}+\mathrm{animal}+\mathrm{e},$$

where each term is as defined in the previous model. The number of ROH was analyzed with a model that included the fixed effect of the breed. Generalized mixed linear model analysis was performed using the SAS PROC GLIMMIX (SAS Inc, 2011).

The relationship between the occurrence of an SNP in a ROH and the type of buffalo was assessed in the ALL_DATA set using a logistic regression model. For each individual, the SNP was coded as 1 if included or 0 if not included in a run. This binary variable was analyzed with the following logistic regression model:

$$log\frac{p}{1-p}={\beta }_{0}+{\beta }_{type},$$

where $p$ is the probability of a SNP to be included in a ROH; ${\beta }_{0}$ is the intercept; and ${\beta }_{type}$ is the fixed effect for the type (river vs swamp). The logistic regression was performed using the SAS PROC LOGISTIC.

## Results

### ALL_DATA set

**Fig. 1:**
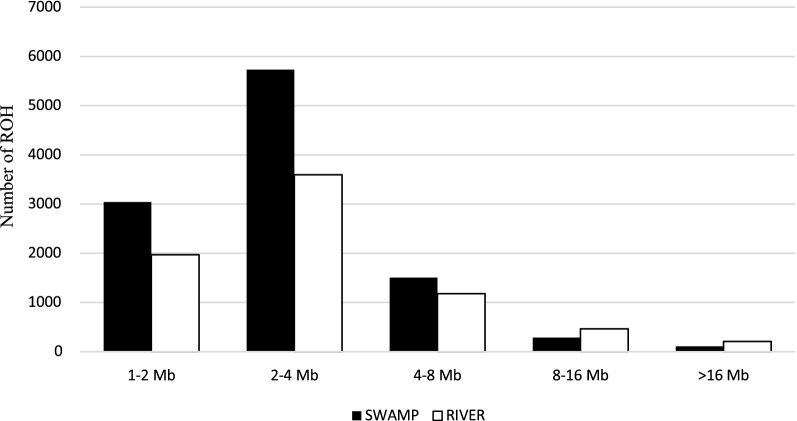
Distribution of length classes of ROH. Frequency distribution of ROH across length classes in river (white bars) and swamp (black bars) buffalo

ROH were found in all breeds and populations for both water buffalo types, with 18,116 ROH detected in the ALL_DATA dataset (Table 2). The largest number (about 59% of the 18,116 ROH) was detected in the swamp buffalo, whereas in river buffalo ROH were longer, but both these statistics varied a lot among populations (Table 2). In the ALL_DATA set, 16,251 ROH were unique. The ROH distribution across length classes had a negative exponential shape (Fig. 1), with the second smallest class, 2 < Mb ≤ 4, being the most abundant in both river and swamp buffaloes. ROH distribution among the chromosomes was proportional to their length for both buffalo types (see Additional file 3: Figure S2).

**Table 2:** Basic statistics of ROH frequency and length for river and swamp buffaloe in the ALL_DATA set

Statistics	River	Swamp	All
Total number of ROH	7429	10,687	18,116
Total number of unique ROH	6712	9539	16,251
Average number of ROH per animal	40 ± 27	70 ± 33	54 ± 33
Average ROH length	4.07 ± 4.47	3.29 ± 3.51	3.60 ± 4.09
Average ROH count per SNP (%)	6.5 ± 3.2	9.4 ± 5.1	7.9 ± 4.5

**Fig. 2:**
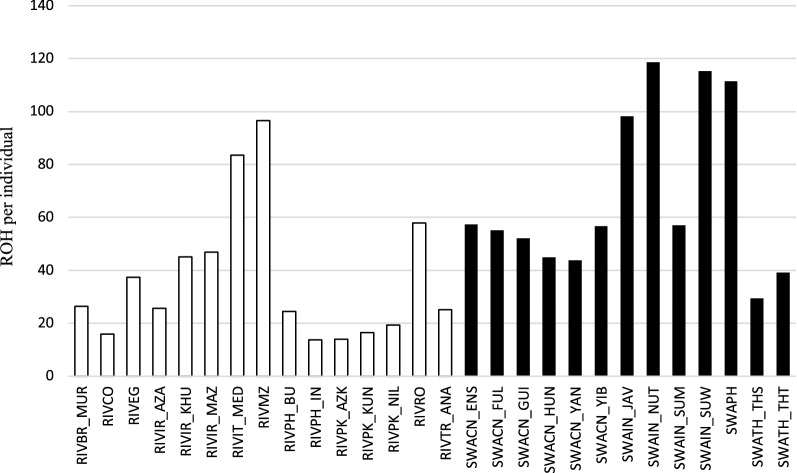
Distribution of number of ROH. Distribution of the average number of ROH per animal in river (white bars) and swamp (black bars) buffalo populations

Swamp populations had more ROH per individual compared to river breeds (Fig. 2). The swamp population of the Indonesian island of Nusa Tenngara of Indonesia had the highest average n_ROH (118.6) and the Indian Murrah river breed had the lowest (13.8). Among river buffaloes, the Mediterranean breed of Mozambique had the highest n_ROH (96.6). These figures were confirmed by the generalized mixed linear model analysis. The average n_ROH was significantly affected by the type and the breed within type, with swamp buffaloes having the highest values (LSMean and SE 60.46 ± 1.24 vs 29.11 ± 0.61) compared to river buffaloes.

**Fig. 3:**
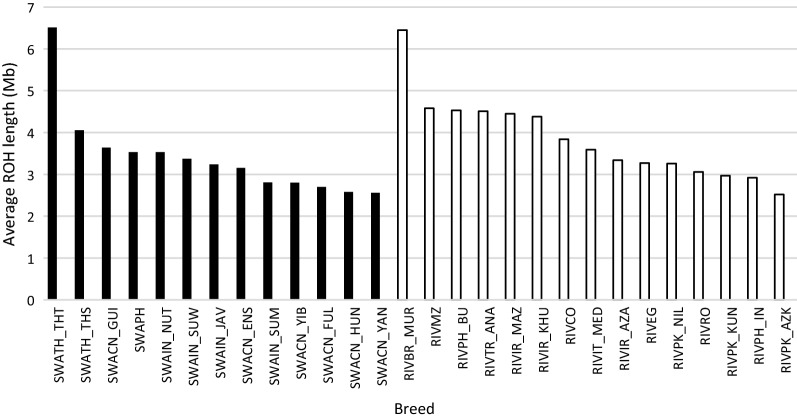
Distribution of length of ROH. Distribution of the average ROH length in river (white bars) and swamp (black bars) buffalo populations

Average l_ROH was generally larger in river buffalo breeds (Fig. 3), although the Thailand swamp population had the greatest average length (6.5 Mb). The longest ROH (52.5 Mb and 354 SNPs, respectively) was detected on *Bubalus bubalis* chromosome (BBU) 10 in an Indonesian Nusa Tenngara individual. The chromosomes with the largest average l_ROH were BBU18 in river (5.8 Mb) and BBU13 in swamp (4.1) buffalo, respectively (see Additional file 4: Figure S3). Type, breed within type, and chromosome significantly affected the l_ROH (*P* < 0.001). The average values of l_ROH were largest for river buffaloes (LSmean and SE Mb 2.87 ± 0.05) compared to swamp buffaloes (2.67 ± 0.04). Significant differences were also observed among chromosomes, breeds or populations and between types (data not shown). LSmeans was largest on BBU18 (3.32 ± 0.11) and smallest on BBU19 (2.33 ± 0.05). The repeatability for this trait was 0.08.

**Fig. 4:**
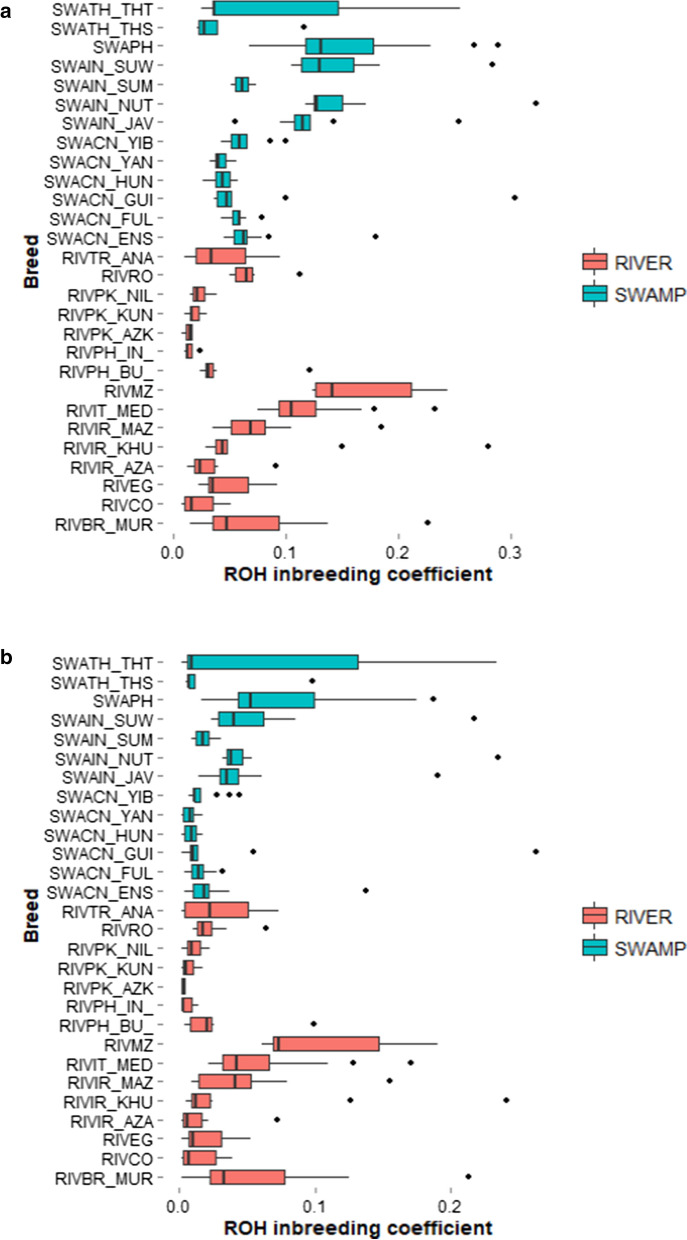
Box-plot of ROH-based inbreeding. Box plot of the ROH-based inbreeding coefficient in different populations/breeds of river (red) and swamp (green), calculated using all the detected ROH (**a**) or ROH longer than 4 Mb (**b**)

Different F_ROH_ values were obtained for the two buffalo types when all homozygous segments were considered (Table 3). Swamp populations tended to have higher F_ROH_ with the highest values observed in the buffaloes from Indonesia, Thailand, and the Philippines (Fig. 4a). Chinese swamp buffaloes showed less variable F_ROH_ values compared to other swamp populations. Among river buffaloes, F_ROH_ was highest for the Mediterranean breed sampled in Mozambique (Fig. 4a) and lowest for those from Pakistan, which also showed the least variability in F_ROH_. When only ROH longer than 4 Mb were used in the calculation, the levels of genomic inbreeding were similar between swamp and river buffaloes (Fig. 4b). The F_ROH_ pattern among breeds or populations within a type was similar to that observed using all the ROH.

**Table 3:** ROH-based coefficient of inbreeding in the two types of river buffalo calculated from the ALL_DATA set using different minimum thresholds of ROH length

Inbreeding coefficient	River buffalo	Swamp buffalo
F_ROH_ all	0.06 ± 0.05	0.09 ± 0.06
F_ROH_ > 2 Mb	0.06 ± 0.05	0.08 ± 0.06
F_ROH_ > 4 Mb	0.04 ± 0.04	0.04 ± 0.05
F_ROH_ > 8 Mb	0.03 ± 0.04	0.03 ± 0.05

The most frequent ROH, i.e. detected in nine animals, was located on BBU16 between 81.6 and 84.4 Mb and was shared by individuals of both river and swamp buffaloes (see Additional file 5: Table S2). Another ROH located on BBU10 at 101 Mb was also shared by both river and swamp buffaloes. Other frequent ROH were found in Chinese swamp buffalo populations, and in Italian and Mozambique river buffalo breeds. It is interesting to note that two ROH located on BBU2 at 49.1 and 50.8 Mb and shared by Chinese and Indonesian swamp buffalo populations, overlapped largely with a third ROH located at 46.2 Mb on BBU2 (see Additional file 5: Table S2).

**Fig. 5:**
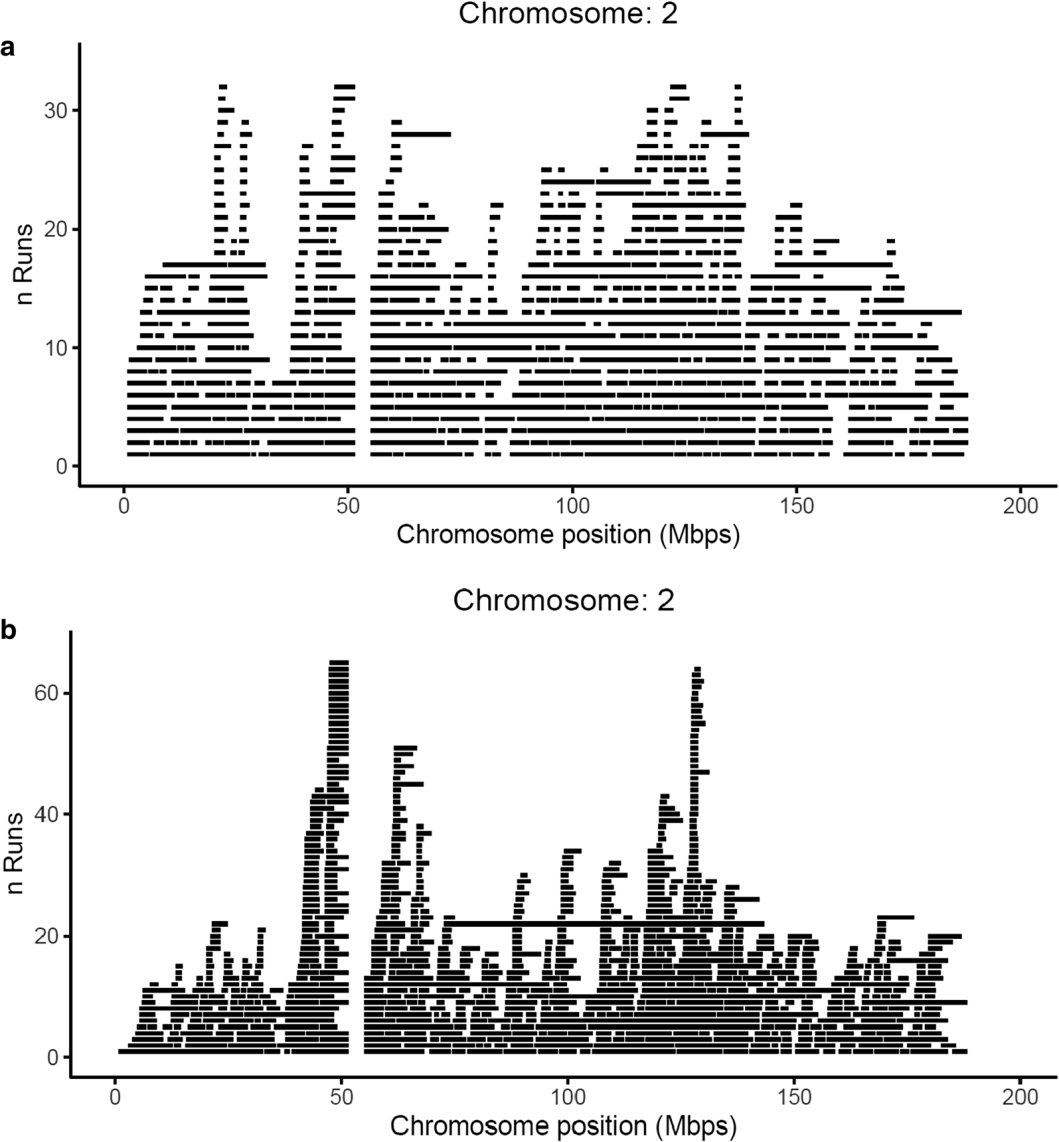
Stacked bar graph of ROH distribution on BBU2. Stacked bar graph of ROH distribution on BBU2 in river (**a**) and swamp (**b**) buffalo

The ROH count per SNP was larger in swamp than in river buffaloes. The largest SNP_ROH_ value (34%, 118 out of 338, i.e. 82 and 36 in swamp and river buffalo, respectively) was detected on BBU2 at about 50.8 Mb. In total, 176 SNPs, located on six chromosomes, exceeded the 99^th^ percentile threshold of the SNP_ROH_ distribution and were considered as significant (Fig. 5). These most significant SNPs clustered in specific regions (Table 4), which were considered as ROH islands. The largest regions were located on BBU1 and BBU2. The functions of the genes that map to these regions were further investigated. Plotting ROH against chromosome position clearly shows the ROH islands that are shared by the two buffalo types: examples for BBU2, 4, and 19 are shown in Fig. 5 and (see Additional file 6: Figure S4 and Additional file 7: Figure S5), respectively.

**Table 4:** Average F_ST_, odd ratio and SNP_ROH_ for the markers in the highlighted ROH islands

ROH island				Average FST		Odds ratio	SNP_ROH_	
BBU	SNP	Start (Mb)	End (Mb)	ROH island	BBU		RIV	SWA
1	20	11.01	12.43	0.46 ± 0.06	0.23 ± 0.10	9.98 ± 2.67	8.10	46.60
1	21	112.56	115.20	0.49 ± 0.06	0.23 ± 0.10	5.27 ± 0.92	9.45	35.70
2	39	46.77	57.78	0.44 ± 0.13	0.22 ± 0.10	3.38 ± 1.07	16.90	40.40
2	7	61.45	62.32	0.40 ± 0.08	0.22 ± 0.10	3.10 ± 0.53	11.80	28.50
2	9	120.40	121.17	0.32 ± 0.07	0.22 ± 0.10	1.99 ± 0.24	14.80	25.00
2	18	127.15	128.80	0.36 ± 0.13	0.22 ± 0.10	3.22 ± 0.55	12.20	31.80
4	13	114.43	117.05	0.28 ± 0.05	0.22 ± 0.08	2.02 ± 0.21	14.70	25.40
7	11	53.00	53.70	0.26 ± 0.02	0.20 ± 0.07	0.74 ± 0.05	21.60	16.70
17	13	50.71	51.62	0.34 ± 0.02	0.22 ± 0.09	1.63 ± 0.46	16.50	24.00
19	20	51.52	52.86	0.36 ± 0.05	0.23 ± 0.08	1.70 ± 0.39	18.00	27.40

SNP_ROH_ = ROH count per SNP

The Manhattan plot of the pattern of LOWESS-corrected F_ST_ values (see Additional file 8: Figure S6) shows strong signals on BBU1, 2, 5, and 8, although 87 SNPs located on 18 chromosomes exceeded the threshold of three standard deviations from the mean. Of these 87 SNPs, 18 were located within ROH islands defined by SNP_ROH_ (Table 4). Four of these SNPs were located between 112.98 and 113.65 Mb on BBU1, 11 between 46.91 and 50.04 Mb on BBU2 and three between 127.85 and 127.96 on BBU3. The average F_ST_ of SNPs located within these ROH hotspots was higher than the average value of the corresponding chromosome (Table 4). Such differences were largest for the ROH islands located on BBU1 and for the first two on BBU2.

The results of the logistic regression highlighted significant differences (P < 0.01) between river and swamp buffaloes in terms of the probability of a SNP to be included within a ROH (*P*_RSNP_ROH_) for 1876 of 17,784 SNPs. The overall average odds ratio (OR) was 1.73 (± 1.37), which indicates that the *P*_RSNP_ROH_ increases by about 70% from river to swamp buffalo. If only the 176 significant SNPs exceeding the 99th percentile of the SNP_ROH_ distribution are considered, the average OR increases to 4.16 (± 1.37), with a fourfold increase of *P*_RSNP_ROH_ in swamp compared to river. Although for most of the 176 significant SNPs the OR was higher than 2, which means that *P*_RSNP_ROH_ is more than twice as high in swamp than in river buffalo, 42 of these SNPs exhibited values lower than 1.0, which indicates that, for these markers, the *P*_RSNP_ROH_ is higher in river than in swamp buffalo. Finally, all ROH islands except one showed average OR values higher than 1 (Table 4). The only ROH island that had an average OR lower than 1 was on BBU7.

### RIVER_DATA set

In total, 19,760 ROH were detected in the RIVER_DATA set, most of which (> 90%) were unique (Table 5). The distribution of ROH across different length classes showed a negative exponential shape, but the most abundant class corresponded to the shortest length (Table 5). The number of ROH was largest in the Italian Mediterranean river buffalo breed and smallest in the Pakistani Aza-Kheli breed, although these breeds had the largest and the smallest sample size, respectively. Average n_ROH (219.86) was highest for the Mozambique breed and lowest for the Indian Murrah sampled in the Philippines (49.75). Generalized mixed model analysis confirmed these results. Differences in n_ROH between breeds (P < 0.001) were significant with the largest LSmeans (95.87 ± 9.70) for the Mozambique buffaloes and the smallest (13.38 ± 2.07) for the Pakistani Aza-Kheli buffaloes. Most of the pairwise comparisons between breeds were highly significant.

**Table 5:** Basic ROH statistics, and their frequency distribution across length classes in the RIVER_DATA and SWAMP_DATA sets

	RIVER_DATA	SWAMP_DATA
Statistics		
Total number of ROH	19,760	10,581
Total number of unique ROH	18,152	9594
Average number of ROH per animal	107 ± 58	69 ± 32
Average ROH length	2.23 ± 2.61	3.31 ± 3.55
Average ROH count per SNP (%)	9.6 ± 4.7	26.9 ± 17.3
Distribution across length classes		
1–2 Mb	14,068 (71.2%)	2971 (28.1%)
2–4 Mb	3955 (20.0%)	5696 (53.8%)
4–8 Mb	1154 (5.9%)	1509 (14.3%)
8–16 Mb	440 (2.2%)	290 (2.7%)
> 16 Mb	143 (0.7%)	115 (1.1%)

The longest average l_ROH was found in the Brazilian Murrah breed (3.22 Mb) and the shortest was in the Pakistani Aza-Kheli breed (1.53 Mb). The general pattern was similar to that observed in the ALL_DATA set. l_ROH was significantly affected by breed and chromosome (*P* < 0.001), with the largest value found for the Brazilian Murrah breed (LSmean and SE Mb 2.07 ± 0.05) and the smallest for the Pakistani Aza-Kheli breed (1.40 ± 0.12). Pairwise comparisons highlighted significant differences between the Brazilian Murrah and all of the other breeds, except the Iranian Mazandarani and Mozambique breeds. BBU5 had the largest l_ROH LSmean (1.82 ± 0.04) and BBU21 the smallest (1.56 ± 0.04). BBU2 was statistically different from BBU12, 17, and 19. The repeatability for this ROH characteristics was 0.05.

The river buffalo individual with the largest number of ROH (274) belonged to the Mediterranean Mozambique breed and that with the smallest number (37) belonged to the Pakistani Aza-Kheli breed. The longest (66.47 Mb) and shortest (1 Mb) ROH were both found in Italian Mediterranean buffaloes, on BBU10 and BBU7, respectively. The most frequently detected ROH, i.e. in nine animals of the Italian and Mozambique Mediterranean breeds, was located on BBU9 (see Additional file 9: Table S3). Four of the five most frequently shared ROH were found in the Mediterranean Italian river buffalo breed.

Inbreeding coefficients for river buffalo, calculated using different sets of ROH (Table 6), were similar to those obtained with the ALL_DATA set (Table 3), except for the value obtained when all ROH were used. The average inbreeding coefficient calculated using all ROH was largest for the Mediterranean Mozambique breed and lowest for the Pakistani breeds. The individual with the largest inbreeding coefficient (0.31) was an Iranian Khuzestani buffalo.

**Table 6:** ROH-based coefficient of inbreeding in the two types of buffalo calculated from the RIVER_DATA and SWAMP_DATA sets using different minimum thresholds of ROH length

Inbreeding coefficient	River	Swamp
F_ROH_ all	0.09 ± 0.06	0.09 ± 0.02
F_ROH_ > 2 Mb	0.05 ± 0.05	0.08 ± 0.06
F_ROH_ > 4 Mb	0.03 ± 0.04	0.04 ± 0.05
F_ROH_ > 8 Mb	0.03 ± 0.03	0.03 ± 0.05

The average SNP_ROH_ in the RIVER_DATA set was 17 (± 8.7). In total, 432 SNPs exceeded the threshold of the 99^th^ percentile of the SNP_ROH_ distribution. They were located on 16 chromosomes (see Additional file 10: Table S4) and clustered into 23 ROH islands with a length ranging from 0.03 to 10.77 Mb. The ROH island detected on BBU2 between 49.7 and 54.8 Mb largely overlapped with that observed in the ALL_DATA set.

### SWAMP_DATA set

The number of ROH detected in swamp buffaloes was about half that found in river buffaloes (Table 5), although it should be noted that the SWAMP_DATA set comprised only about one-third of the SNPs in the RIVER_DATA set. The SNPs included in the SWAMP_DATA set overlapped with those in the ALL_DATA set with a few exceptions due to independent pruning for missing data and MAF. For this reason, the results for ROH number and ROH features in swamp buffaloes obtained with the two datasets were very similar (Tables 2, 5). The Philippine swamp buffalo population had the largest number of ROH (2322) and the Thailand population had the longest average ROH length (2.35 Mb). The second shortest class of ROH was predominant in the swamp type with about 54% of the homozygous segments being in the 2 < Mb ≤ 4 class length (Table 5). The most frequently detected ROH (see Additional file 10: Table S4), i.e. in seven animals, were located on BBU1 at 11.0 Mb and BBU2 at 50.8 Mb.

l_ROH was significantly affected by both population and chromosome (P < 0.001). The swamp buffalo population of Thailand had the largest LSmean, which was statistically different from that of most of other groups (Mb 3.55 ± 0.21), and the Chinese Hunan swamp buffalo (Mb 2.35 ± 0.10) had the smallest LSmean. The repeatability of this ROH characteristic was 0.08. n_ROH was affected by population, with the largest (118.23 ± 8.64) and smallest (28.57 ± 2.48) LS means found for Indonesian Nusa Tenggara and Thailand buffaloes, respectively. Pairwise comparisons mostly highlighted significant differences between Indonesian buffaloes and the other populations.

The Indonesian Nusa Tenggara population had the largest average inbreeding coefficient (0.16 ± 0.07) and the Chinese Yangzou population had the smallest (0.04 ± 0.01). The individual with the largest F_ROH_ (0.32) was a Nusa Tenggara buffalo.

The average SNP_ROH_ in the SWAMP_DATA set was 15 (± 7.8). One hundred and sixty-nine SNPs exceeded the threshold of the 99th percentile of the SNP_ROH_ distribution and were located on six chromosomes (see Additional file 10: Table S4). Fourteen ROH islands were detected, ranging in size from 0.03 Mb (2 SNPs) on BBU17 to 6.77 Mb (and 33 SNPs) on BBU1. The three significant SNPs located on BBU8 were not considered because they were separated by more than 1 Mb from each other. Most of the ROH islands detected in the SWAMP_DATA set coincided with those found in the ALL_DATA set and, to a lesser extent, in the RIVER_DATA sets. The ROH island, located between 49 and 57 Mb on BBU2 was common across all three datasets.

### Gene function analysis

Because the ROH identified in the SWAMP_DATA set overlapped with those in the ALL_DATA set, we investigated the functions of the genes located in ROH islands for the ALL_DATA and RIVER_DATA sets only.

### ALL_DATA set

Two large ROH hotspots were detected on BBU1 (Table 4), which harbor several annotated genes (see Additional file 11: Table S5). Four of the genes located in the first ROH (between 11.01 and 12.43 Mb) are associated with reproduction traits in several livestock species [36–39]: *ADAM32*, *ADAM9, PLEKHA2* and *FGFR1.* The latter was also found within a ROH detected in Hanwoo, Black Angus and Holstein cattle [22]. The second largest ROH island on BBU1, located between 112.6 and 115.2 Mb, also contains genes that have a role in livestock reproduction, i.e., *ADCY2*, *KALRN,* and *IQCG* [40]. The ROH island on BBU1 also contains the *UMPS* gene, which has been reported to be associated with feed efficiency in beef cattle [41].

The large ROH island located between 46.8 and 57.8 Mb on BBU2 (Table 4) contains many annotated genes. Four of these genes are associated with reproduction traits: *DST* [42, 43], *RAB23* [44], *KHDRBS2* [45–47], and *TUBGCP5* [48]. The ROH island on BBU2 also contains three genes that are involved in skin pigmentation and eye disorders: *LGSN* [49], *OCA2* [50, 51], and *HERC2* [52] and have been identified in selection signatures in buffalo [25] and in cattle [51]. Additional genes that map to this region are: *PTPN18*, a gene involved in the regulation of the neuronal leptin and insulin signaling pathways [53] and is associated with feeding behavior in pigs [54], and *AMER3*, which is involved in embryogenesis [55]. This ROH island on BBU2 also harbors the *ARHGEF4* gene, which is associated with milk production traits in dairy cattle [56] and the *PLEKHB2* gene, which is associated with residual feed intake in beef cattle [57]. Both the *AMER3* and *PLEKHB2* genes have been reported to be located in a ROH island on chromosome 18 in the Lipizzan horse [58].

No candidate genes were found in the other ROH islands detected in the ALL_DATA set.

### RIVER_DATA set

The largest ROH island in the RIVER_DATA set (about 10.8 Mb and 109 SNPs) was detected on BBU3 (see Additional file 10: Table S4). We identified three genes related to environmental adaptation and body development, i.e., *HLF*, *MMD*, and *STXBP4*, in this region (see Additional file 11: Table S5). These genes have also been found in a ROH island located on horse chromosome 11 [59]. The second largest ROH island in river buffalo is located on BBU1. It contains genes that are associated with muscle contraction (*MYOM2*) [60], iron content (*RCAN1*) [61], and fatty acid metabolism (*ARHGEF10*) [62]. Further interesting genes that map in this ROH island are *ARHGEF10, CLN8* and *DGAP2*, that were associated with hairless phenotype in pigs [63]. This ROH island also contains a group of genes that are involved in polledness in cattle [64, 65] and yak [66]: *IFNGR2*, *IFNAR1*, and *SYNJ1*.

Several other interesting genes were identified within the ROH islands detected in river buffaloes: *LGR5* on BBU4, previously proposed as a candidate gene for supernumerary teats in cattle [67]; *UBE2H* on BBU8 that is associated with feed efficiency in cattle [68]; two genes on BBU9: *PCDHB7,* which has been suggested as a candidate gene for milk protein composition [69], and CD14, which is involved in the immune response of the cattle mammary tissue infected by *Streptococcus agalactiae* [70]; and *CDK10* on BBU18 at ~ 14–14.6 Mb, which has been reported to be present in a selection signature in African local cattle breeds [71].

### Principal component analysis of swamp and river G_ROH_ matrices

**Fig. 6:**
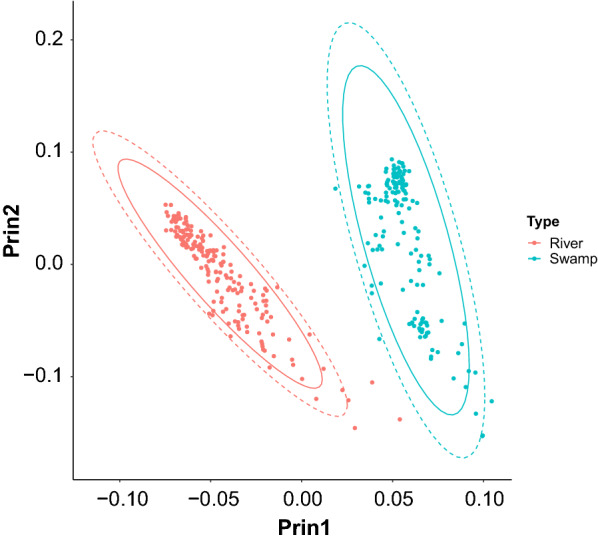
Eigenvectors on ROH-based genomic relationship matrix. Plot of the first two eigenvectors of the ROH-based genomic relationship matrix calculated using SNPs coded according to their occurrence in ROH longer than 4 Mb (red = river; turquoise = swamp). Solid line indicates the 95% confident interval; dotted line indicates the 99% confident interval

**Fig. 7:**
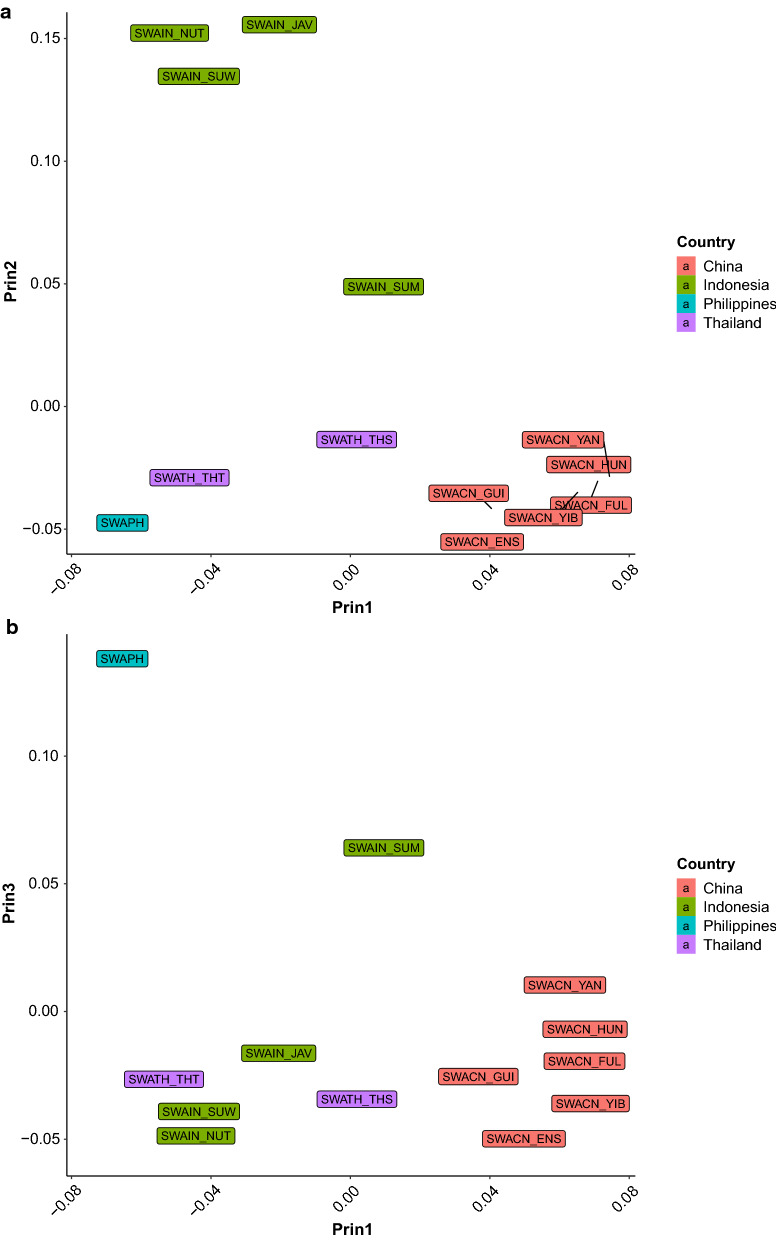
Eigenvectors on ROH-based genomic relationship matrix in swamp buffaloes. Plot of the swamp buffalo population means of the first two (**a**), and the third and second (**b**) eigenvectors of the ROH-based genomic relationship matrix calculated using ROH longer than 4 Mb. Different colors indicate different countries of origin

**Fig. 8:**
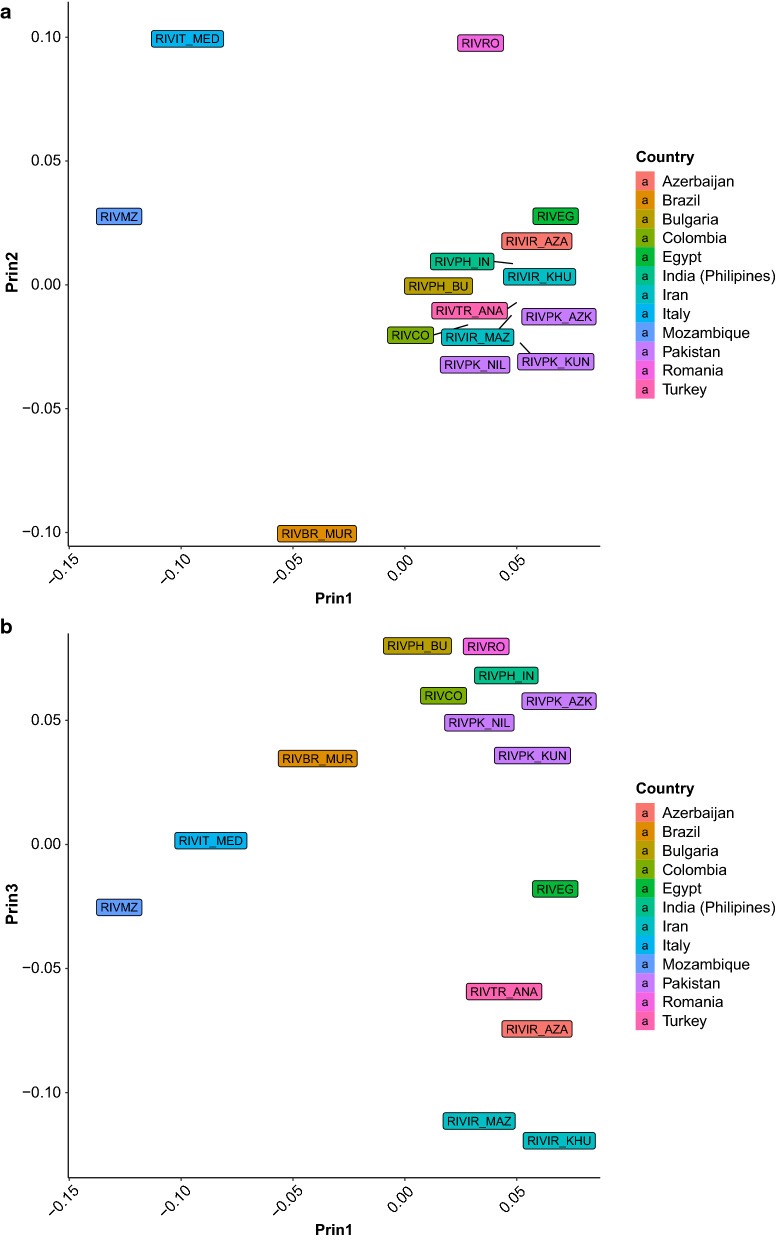
Eigenvectors on ROH-based genomic relationship matrix in river buffaloes. Plot of the river buffalo population means of the first two (**a**), and the third and second (**b**) eigenvectors of the ROH-based genomic relationship matrix calculated using ROH longer than 4 Mb. Different colors indicate different countries of origin

The first two eigenvectors of the **G**_**ROH**_ calculated by using the SNPs of the ALL_DATA set, explained 4 and 1% of the total variance, respectively. The plot of eigenvector coefficients (Fig. 6) shows a clear distinction between the two buffalo types. The first eigenvector separates river and swamp buffaloes, and the second one shows a within-type gradient. The differences between populations and breed are better illustrated on the principal component analysis that was carried out on the **G**_**ROH**_ derived from the RIVER_DATA and SWAMP_DATA sets, respectively. Moreover, the patterns are easier to detect if the population means of eigenvectors are plotted, instead of the coefficients for each single animal (Figs. 7a, b, 8a, b). In this case, swamp buffalo populations display a geographical North–South cline along the second eigenvector (Fig. 7a), with the Indonesian populations clustered at the top left, with the exception of the Sumatran population which is in an intermediate position, and the Chinese populations at the bottom right of the plot, respectively. The first eigenvector clusters the Chinese populations and separates them from Indonesian and Thailand buffaloes. The third eigenvector (Fig. 7b) emphasizes the distance between swamp buffaloes from the Philippines and, the Indonesian populations.

The pattern among river buffalo breeds is less well-defined, but it also follows partly a geographical distribution, with an East–West orientation of the first eigenvector (Fig. 8a) that clusters the breeds of the eastern and middle east countries on the right side of the plot, and the two Mediterranean breeds, Italian and Mozambique, in the top left, respectively. The second eigenvector distinguishes the Mediterranean and Iranian breeds. Of interest, the third eigenvector (Fig. 8b) shows a separation between breeds from Pakistan, Egypt, Turkey, and Iran.

## Discussion

### ROH distribution

Analysis of ROH distributions in river and swamp buffaloes, and between populations/breeds provide interesting insights into their genetic structure. ROH were detected in both buffalo types and in all breeds/populations. The number of ROH was larger in swamp (> 43%) than in river buffaloes when they were analyzed jointly using the same set of SNPs. The average ROH frequency per animal detected in the two buffalo types (40 in the river and 77 in the swamp, respectively) is comparable with figures that have been previously reported for buffalo, e.g., Aza-Kheli and Khuzestani Iranian river buffalo breeds that have an average n_ROH of 21 and 33, respectively [24]. The average ROH frequency per animal in other livestock species ranges from 24 to 77 in sheep and goats [72–74], from 40 to 90 in *Bos taurus* cattle [21, 27, 75] and 55 in *Bos indicus* cattle [76]. However, the number of ROH detected depends on the density of the SNP panel, the informativeness and size of the sample, and on the algorithms and ROH parameters used for ROH identification. This is confirmed by our results, which show that different numbers of ROH were detected for the same populations depending on the ALL_DATA or RIVER_DATA set used, which differ in the number of SNPs.

The occurrence of ROH in a population mirrors its genetic and demographic history. The present study highlighted a difference in ROH number between river and swamp buffaloes, especially for the shortest ROH (< 4 Mb) (Fig. 1). Swamp buffalo individuals showed both a larger number of homozygous segments and a larger ROH coverage of the genome compared to river buffaloes. The conclusions based on descriptive statistics are supported by the results of the logistic regression analysis that highlighted a significantly higher probability of a SNP to be included in a ROH in swamp than in river buffalo. The effect of ascertainment bias of the SNP array was, at least in part, addressed by using the 17.8 K SNPs that were polymorphic in swamp buffalo. This reduced set of SNPs showed substantial concordance of results with the RIVER_DATA set that included 46 K SNPs, which suggests that the lower density SNP panel was sufficient to identify coarse features of the buffalo genome structure. In a previous study, a subset of 20 K SNPs from the same beadchip used in this study has been used to differentiate swamp buffalo from river buffalo [77]. As a general consideration, it should be pointed out that short ROH are more likely to be false positives than long ROH. Some authors pointed out that the use of 50 K SNP panels may overestimate the number of ROH shorter than 5 Mb because of the long gap between homozygous SNPs [75, 78]. In our paper, the average distance between SNPs in the shortest ROH class was similar for both buffalo types, 90 kb (1–2 Mb class) and 135 kb (2–4 Mb class), respectively. When the ROH shorter than 4 Mb were discarded, the pattern of ROH and ROH genome coverage remained essentially unchanged (see Additional file 12: Figure S7, Additional file 13: Figure S8), with most of the river buffalo individuals showing a smaller number and a reduced ROH genome coverage compared to the swamp buffalo individuals.

The observed higher frequency of short ROH in swamp buffalo individuals is likely to be related to their genetic history, in particular to the lack of strong anthropogenic selection and to geographic differentiation [77]. The river buffalo type has been subjected to more intense selection than the swamp type, especially for dairy traits [7, 10, 15]. In our study, the average ROH length was longer in river buffalo when the two buffalo types were analyzed using the same set of SNPs. This difference was confirmed by the generalized mixed model analysis that also identified differences between breeds or populations, showing a significant within-type heterogeneity. Variation in ROH length as a result of different selection pressures has been reported in cattle, in which it has contributed to the maintenance of long homozygous tracts [19]. A higher frequency of short ROH (< 4 Mb) has been reported in beef compared to dairy cattle [27], which was attributed to the more intense selection in dairy breeds. A worldwide analysis of homozygosity patterns in goats reported a larger proportion of the genome covered by ROH in populations farmed on islands, due to the geographical isolation, and for local breeds, compared to globally used breeds [68]. A relationship between length of homozygous segments and geographical differentiation has also been observed in humans, e.g., an excess of shorter ROH was found in populations from the Pacific Ocean islands [18]. The small population sizes and geographical isolation on these islands were proposed as possible explanations for these results.

### Dissection of the G_ROH_

Genetic stratification of populations can be detected from eigenvalue analysis of genomic relationship matrices. The eigenvalue analysis of the standard SNP-based genomic matrix ($\mathbf{G}$) for domestic water buffalo showed a clear separation between swamp and river buffalo types [7]. A ROH-derived genomic matrix may capture different aspects of the relationships between individuals, based on the sharing of homozygous segments. Luan et al. [20] developed an identical-by-descent (IBD) **G**_**ROH**_ for genomic prediction purposes based on the coalescence theory and was able to account for mutation and recombination [79]. In our paper, a method for constructing an identical-by-state (IBS) **G**_**ROH**_ based on the recoding of SNP genotypes according to their location within a ROH is proposed. This method estimates a matrix that could be calculated following the method of [31]_._ The separation between types and breeds using the eigenvalue decomposition of the **G**_**ROH**_ matrix that is calculated by using ROH longer than 4 Mb (Fig. 6) gives a pattern similar to that obtained from a standard genomic relationship matrix using individual SNPs (see [7]), including the North–South cline exhibited by swamp buffalo populations (Fig. 7a). Geographical differentiation of many populations based on ROH analyses has been reported for a very wide range of mammalian species [23, 28, 80], especially when unselected or local populations were investigated. The clustering of the Chinese swamp buffalo populations and their distance from Indonesian and Philippine individuals confirms previous reports based on the analysis of microsatellite loci [81] that indicated a low degree of genetic differentiation in Chinese swamp buffaloes compared to south-east Asian animals. The large distance between Indonesian buffaloes and the other populations (Fig. 7a) may be explained by the effect of geographical isolation and genetic drift. The intermediate position of the swamp buffalo population from Sumatra agrees with the results of [7] who reported that only 30% of the genome was shared between Sumatra and the other Indonesian populations. The clear separation between swamp buffaloes from the Philippines and the other swamp buffalo populations based on the third principal component of the **G**_**ROH**_ (Fig. 7b) can be explained by the introgression of river buffalo gene pools through crossbreeding that is used to improve milk production [7]. Such crossbreeding between the two buffalo types in the Philippines was initiated nearly 50 years ago [82] and introgression of river buffalo genes into the genome of swamp buffaloes is currently increasing.

The distribution of river buffalo breeds does not follow a strict geographical pattern. Figure 8a, b show that Iranian, Turkish, and Egyptian river buffaloes cluster together, in agreement with their geographical proximity, whereas breeds from Italy, Mozambique, and, to a lesser extent, Romania, and Brazil are separated from the other groups. This can be explained by the population histories, i.e., river buffaloes from Italy have been exported to Mozambique, Brazil [82] and Romania, and hence these populations share a common “Mediterranean” genomic background. Murrah buffaloes from India have also been imported into Brazil [82, 83]. Of interest is the relative proximity between the Romanian river buffalo and the Bulgarian Murrah river buffalo (Fig. 8b). The Bulgarian breed originated from crosses between indigenous Mediterranean animals and imported Indian Murrah buffaloes [7]. Additional crosses with Romanian river buffaloes have also been reported [84]. Thus, the first principal component of the **G**_**ROH**_ of the river buffalo breeds could be interpreted as an index of the “Mediterranean” content of the genome, whereas the second dimension is an index of the transition between Mediterranean and Murrah buffalo genomes.

### ROH-based inbreeding

Traditionally, estimation of inbreeding has been based on pedigree data, which often underestimated the true level of inbreeding because, usually, pedigree data are only available for a few generations [85]. Calculation based on SNP data is more accurate and it enables genomic relationships to be estimated even when pedigree records are not available or incomplete, as in the case of buffaloes. In the present work, values of F_ROH_ varied greatly between populations, especially for the swamp buffaloes, and depended on the minimum length of the ROH considered in the calculation. Higher inbreeding coefficients were obtained using all the ROH in the calculation. Mean values of F_ROH_ calculated in the present study for Brazilian Murrah, Aza-Kheli and Khuzestani breeds (Fig. 4a) are close to those reported in literature for these three breeds (i.e., 2%) by [25].

The use of longer ROH (> 4 Mb) gave an average F_ROH_ of 3 to 4% for both types. Iranian and Chinese breeds had the lowest values and the least variability in F_ROH_ for the river and swamp buffalo types, respectively. The F_ROH_ variability could be due to differences in genetic history, breeding management, and/or sampling effect among populations. For example, river buffaloes sampled in Mozambique, that exhibited a large F_ROH_ variability (Fig. 4), derive from a well-known exportation of Mediterranean buffaloes from central Italy in 1969 [82]. Likely due to a founder effect and the subsequent prolonged isolation, the current population of river buffaloes from Mozambique displays the lowest values for both observed and expected heterozygosity values among all river buffalo populations [7], which is likely due to a founder effect and subsequent prolonged isolation. Genomic inbreeding coefficients obtained in our study using ROH longer than 4 Mb are generally slightly higher than pedigree-based values reported in the literature. Values between 1.2 and 2.4% were reported for Brazilian Murrah and Mediterranean buffaloes farmed in Brazil [83, 84], respectively. Inbreeding coefficients of 2.4 and 3.42% have been reported for buffalo populations from the Mediterranean area [85] and Iranian buffaloes [86], respectively.

A higher inbreeding level is expected for more intensely selected animals, i.e., from the river buffalo type. However, we observed a substantial similarity between the two buffalo types in the ALL_DATA set, except when all ROH were considered (Table 3). The higher F_ROH_ observed for swamp buffaloes in this case was basically due to a larger n_ROH, and particularly of short length (< 4 Mb) (Table 2; Fig. 1). Such a difference between the two buffalo types could have biased downwards the inbreeding estimation in river buffaloes. However, the results of the analysis carried out on the RIVER_DATA set with many more SNPs (about 2.5 times more) showed that, in spite of a larger number of ROH per individual (107 vs 40), the F_ROH_ values for river buffalo were quite close to those obtained with the ALL_DATA set. The effect of this more intense selection in river buffalo on ROH features is essentially observed for ROH length, which is longer for river than swamp buffaloes, and not for the ROH based genomic inbreeding. Such a moderate effect on the structure of the river buffalo genome should be ascribed to the lower selection pressure that this species has been subjected to in comparison with specialized dairy cattle breeds. Zhang et al. [87] pointed out that organized buffalo breeding programs that use artificial insemination still need to be developed in many countries.

### ROH islands

Genomic regions characterized by a high level of homozygosity have been detected in many livestock species [24, 28, 58, 72, 74, 76]. ROH occurrence may be the result of common ancestry, selection pressure, chromosome structure, and linkage disequilibrium [78]. In our work, we detected ROH that were shared among different buffalo populations. In particular, 10 unique ROH that started and finished at exactly the same positions, were found in six or more individuals from different populations. A ROH on BBU16 occurred in six river buffaloes of breeds from Italy and Mozambique, which are known to be genetically related. In cattle, shared ROH have been detected in local breeds from the two main Italian islands, Sicily and Sardinia [28]. The same ROH were found in genetically related populations, but not in a cosmopolitan breed [28]. Some of the ROH detected in our study were present in both swamp and river buffaloes. Crossbreeding between the two buffalo types to improve swamp buffalo productivity has been a common practice in several east Asian countries [7, 12] and in central America [88]. Transfer and ongoing selection on genomic regions associated with productivity may, in part, explain these shared ROH.

The use of unique ROH to study the genomic similarity between individuals is restrictive when they share the core of a region of homozygosity that does not start or finish exactly at the same position. Since a ROH roughly reflects an IBS haplotype, the precise location of the beginning and end of a ROH may not be important. The use of ROH islands in our study (i.e., a stretch of consecutive SNPs where SNP_ROH_ exceed a certain threshold) is a more flexible way of comparing homozygous regions between individuals. The largest ROH island detected in the two buffalo types, using the three datasets, was located on BBU2, between 47 and 58 Mb. A ROH island located on BBU2 between 52.8 and 53.8 Mb was recently reported in Murrah buffaloes [25]. A ROH hotspot has also been detected at the corresponding position on bovine chromosome 2 [21], which is the homolog of BBU2 [11]. Moreover, ROH islands on chromosome 2 at 68.7, 71.3, and 81.9 Mb have been reported in *Bos indicus* [76]. These ROH harbor genes that are related to dairy traits. A ROH island on *Ovis aries* chromosome OAR2 has been detected in three sheep breeds (Belclare, Suffolk and Texel) [29]. This ROH overlaps the *myostatin* (*MSTN*) gene, which is involved in muscle development and most likely is under selection. A possible interpretation for the occurrence of the same ROH island in different species could be due to evolutionary convergence caused by the selection on the same group of genes. It is noteworthy that two of the genes present in the ROH island on BBU2 (*ARHGEF4* and *PLEKHB2)* which are associated with milk yield and feed efficiency in ruminants, are also present in a ROH hotspot on chromosome 18 of the horse. Although shorter ROH islands represent a weak signal that needs to be carefully validated with other studies and approaches, the consistency between the results on the ROH located on BBU2 with the three datasets of the present study and those from other studies on buffalo and other species suggests that the ROH approach is useful to study the major features of genomes.

Short ROH are ancient and may derive from environmental adaptation, whereas longer ROH are more recent and are more likely to result from artificial selection or population bottleneck events. We found that ROH were longer in river than swamp buffalo breeds, which reflects the different histories and usages of each buffalo type worldwide. In fact, river buffaloes are subdivided in a number of well-recognizable breeds, based on geographical location and morphological traits (e.g., Murrah, Nili-Ravi, Kundi, Jafarabadi and Nagpuri in India and Pakistan, or the Mediterranean buffalo breed group spread in Italy, Bulgaria, Romania, Greece, Turkey, Egypt, Iran, Iraq, and Syria). They are mainly farmed and selected for milk production, and secondarily for meat. Conversely, swamp buffaloes are phenotypically homogeneous throughout their distribution area and no breeds are formally recognized. Swamp buffaloes, which are farmed primarily as draught animals for ploughing and transport, are not subjected to intense human-mediated selective pressures and also provide meat as a secondary product.

A few genes related to production traits were located in the ROH islands detected on the buffalo genome. *ARHGEF4*, which is known to be associated to dairy traits, was detected in the ROH on BBU2 that is shared between both buffalo types. All the other genes related to production traits were identified in the analyses of the RIVER_DATA set but not of the SWAMP_DATA set: e.g., *PCDHB7* and *UBE2H,* which are related to dairy traits, and *TRNAG*, *MYOM2*, *AADAT*, and *RCAN1*, which are related to beef traits. *MYOM2* is present in a signature of selection that was identified in a comparison between Atzeri and Khuzestani Iranian buffaloes using F_ST_ metrics [16]. The small number of genes related to dairy traits that was retrieved in the detected selection sweeps confirms the low selection pressure for milk production in buffalo in comparison, for example, with cattle.

Our results suggest that the main driving force in shaping the genome of the domestic water buffalo is adaptation to the environment. This is supported by most of the genes that map to the ROH islands detected in the ALL_DATA set and that are related to fitness traits. In particular, the largest group consists of genes that are associated with reproduction traits in livestock: male fertility (*ADAM32*, *IQCG*, and *DST*), female fertility (*ADAM9*, *ADCY2*, *KALRN*, *RAB23*, *KHDRBS2*, and *TUBGCP5*) and embryogenesis (*ANXA10*, *PLEKHA2*, *FGFR1*, and *AMER3*). The second largest group of genes identified in ROH are involved in disease resistance such as *KALRN*, *ITGB5*, *MUC13*, *HEG1*, and *LHCR3* in the ALL_DATA set, and *HLF, MMD, SH3RF1, STXBP4*, CD14, and CDK10, in the RIVER_DATA set.

Surviving and producing in arid areas represents a strong environmental challenge for livestock. The buffalo is a species that has a reduced tolerance to heat stress due to the poor distribution of its sweat glands, its very short and sparse hairs, and the dark color of its body [89]. However, in many countries, buffaloes are exposed to extreme heat stress, for example, in Iran [16]. Wallowing in mud and water protects them from solar irradiation and provides a cooling effect; it is a learnt adaptive behavior that buffaloes use to adapt to tropical and subtropical climates [90]. However, natural selection for resistance to heat stress may have occurred in some breeds [24]. The genes identified in the ROH islands in our study included some genes that have been implicated in the mechanisms of tolerance to hot and humid climates, such as those associated with skin pigmentation and eye disorders (*LGSN*, *OCA2*, and *HERC2),* and breathing rate (*LIMS2*). Another interesting group of genes that were identified within ROH is associated with the hairless phenotype (*ARHGEF10*, *CLN8*, and *DGAP2*) [62]. Finally, further evidence of environmental adaptation is suggested by the detection in ROH of genes related to feed efficiency (*UMPS*, *TPN18*, and *PLEKHB2*). The efficiency of the process of digestion is extremely important for surviving in extreme arid areas [91]. Our results agree with previous reports on indicine cattle, which highlighted ROH hotspots that harbor genes involved in the mechanism of adaptation [92].

Selection footprints identified from ROH analyses should be corroborated by other well-proven techniques. Although one should keep in mind that most metrics are based on comparisons between breeds or populations, whereas the rationale behind the search for selection sweeps using ROH is the sharing of homozygous regions by a large number of animals. In our paper, F_ST_ values were calculated for the ALL_DATA set. Some SNPs that distinguished the two buffalo types also frequently occurred in homozygous regions. Moreover, average F_ST_ values of SNPs included in the ROH islands were larger than the average values for the corresponding chromosome. These results further confirm that different metrics, although calculated from the same data, can offer complementary perspectives to analyze genome features and we recommend their combined use.

## Conclusions

In the present study, runs of homozygosity were used to investigate the genomic structure of the two buffalo types and their breeds, and to compare populations of domestic water buffalo. In particular, the dissection of the ROH-based genomic relationship matrix suggested that the two buffalo types have undergone environmental adaptation. The geographical isolation of populations together with genetic drift, have played a substantial role in shaping the genome of buffalos, especially of the swamp type. Analysis of the distribution of ROH islands on BBU2 identified a region that is shared with other ruminant species. The evolutionary significance of this region merits further investigation.

## Supplementary Information

**Additional file 1:** **Table S1.** Minor allele frequency in river and swamp buffalos.

**Additional file 2:** **Figure S1.** Plot of linkage disequilibrium (r^2^) according to the distance between markers in river and swamp.

**Additional file 3:** **Figure S2.** Frequency distribution of ROH across chromosomes in river (black bars) and swamp (white bars) buffalo.

**Additional file 4:** **Figure S3.** Distribution of the average ROH length per chromosome in river (white bars) and swamp (black bars) buffalo populations.

**Additional file 5:** **Table S2.** Top ten most frequently detected ROH in the ALL_DATA set.

**Additional file 6:** **Figure S4.** Stacked bar graph of ROH distribution on BBU4 in river (a) and swamp (b) buffalo.

**Additional file 7: Figure S5.** Stacked bar graph of ROH distribution on BBU17 in river (a) and swamp (b) buffalo.

**Additional file 8:** **Figure S6.** Manhattan plot of smoothed *F*_ST_ values for chromosomes 1 to 24.

**Additional file 9: Table S3.** Top five most frequently detected ROH in RIVER_DATA and SWAMP_DATA sets.

**Additional file 10:** **Table S4.** Chromosomal location of significant SNPs (i.e. with SNP_ROH_ values located in the top 1%) in the RIVER_DATA and SWAMP_DATA sets.

**Additional file 11:** **Table S5.** Genes mapped in the ROH islands highlighted in this study [93–112].

**Additional file 12:** **Figure S7.** Relationship between number of ROH and total length of the genome covered by ROH (using all ROH detected in ALL_DATA set).

**Additional file 13:** **Figure S8.** Relationship between number of ROH and total length of the genome covered by ROH (using ROH with length ≥ 4 Mb detected in the ALL_DATA set).

## Data Availability

SNPs genotype data used in this study are available at the Dryad repository (https://doi.org/10.5061/dryad.h0cc7).
