# Supplementary material for: The distribution of runs of homozygosity in the genome of river and swamp buffaloes reveals a history of adaptation, migration and crossbred events
Source: Genet Sel Evol. 2021 Feb 27;53:20. doi: 10.1186/s12711-021-00616-3 (PMC7912491; doi:10.1186/s12711-021-00616-3)
Supplement: Supplementary file 1 — Additional file 1: Table S1. Minor allele frequency in river and swamp buffalos. [file 12711_2021_616_MOESM1_ESM.docx]

| Chromosome | MAF RIVER | MAF SWAMP |
| --- | --- | --- |
| 1 | 0.338±0.111 | 0.211±0.148 |
| 2 | 0.337±0.113 | 0.217±0.149 |
| 3 | 0.331±0.116 | 0.226±0.15 |
| 4 | 0.339±0.111 | 0.221±0.147 |
| 5 | 0.334±0.113 | 0.222±0.149 |
| 6 | 0.336±0.113 | 0.217±0.147 |
| 7 | 0.339±0.109 | 0.232±0.148 |
| 8 | 0.341±0.11 | 0.225±0.149 |
| 9 | 0.335±0.111 | 0.221±0.147 |
| 10 | 0.334±0.11 | 0.21±0.152 |
| 11 | 0.338±0.109 | 0.21±0.151 |
| 12 | 0.338±0.113 | 0.216±0.148 |
| 13 | 0.335±0.112 | 0.225±0.144 |
| 14 | 0.337±0.11 | 0.216±0.146 |
| 15 | 0.333±0.114 | 0.222±0.146 |
| 16 | 0.33±0.113 | 0.215±0.147 |
| 17 | 0.331±0.115 | 0.217±0.149 |
| 18 | 0.332±0.114 | 0.206±0.145 |
| 19 | 0.336±0.113 | 0.219±0.148 |
| 20 | 0.336±0.111 | 0.219±0.142 |
| 21 | 0.333±0.108 | 0.22±0.15 |
| 22 | 0.342±0.105 | 0.227±0.146 |
| 23 | 0.336±0.11 | 0.212±0.145 |
| 24 | 0.328±0.116 | 0.204±0.147 |
