# Supplementary figures and images for: The distribution of runs of homozygosity in the genome of river and swamp buffaloes reveals a history of adaptation, migration and crossbred events
Source: Genet Sel Evol. 2021 Feb 27;53:20. doi: 10.1186/s12711-021-00616-3 (PMC7912491; doi:10.1186/s12711-021-00616-3)

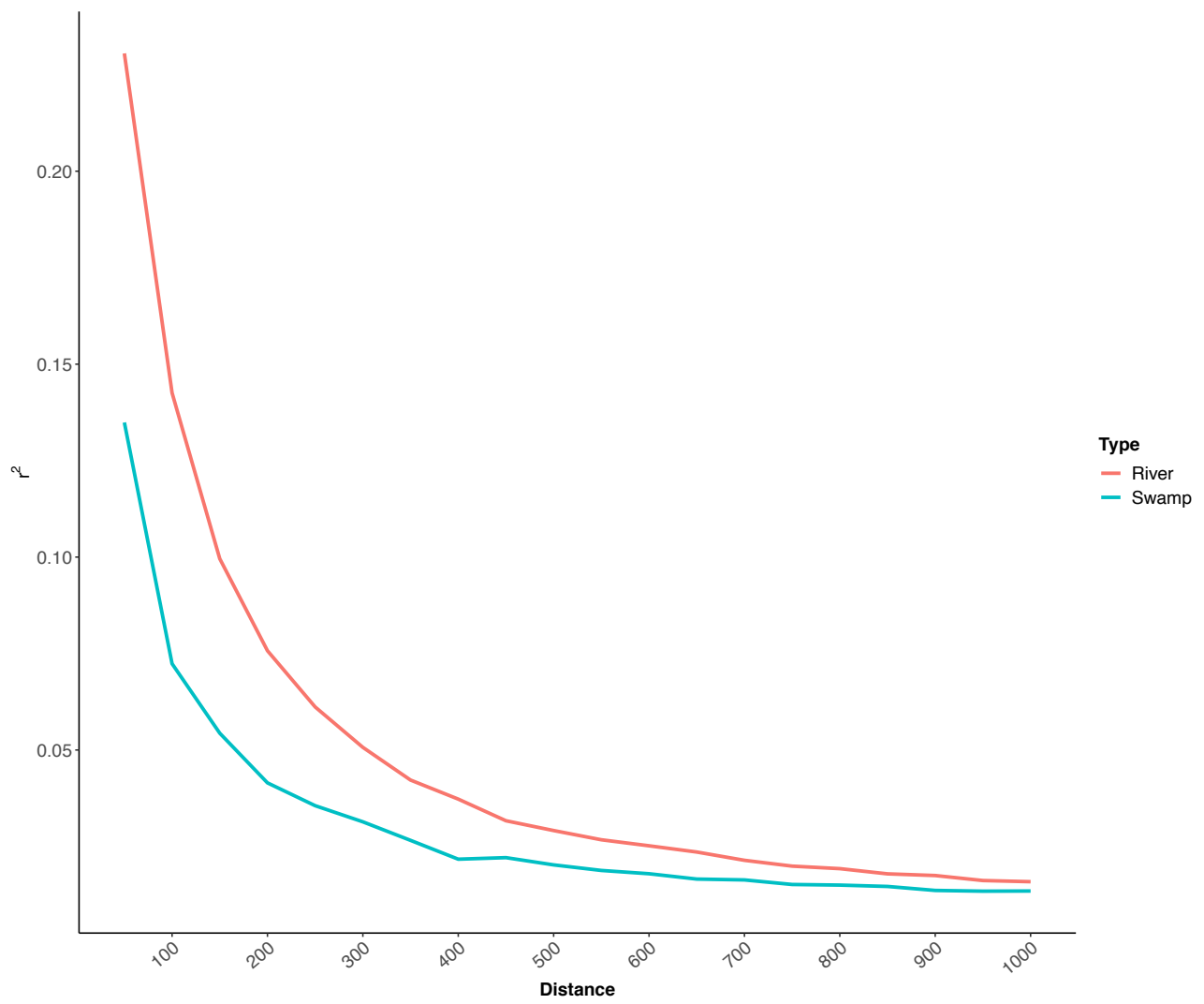

Supplement: Supplementary file 2 — Additional file 2: Figure S1. Plot of linkage disequilibrium (r2) according to the distance between markers in river and swamp. [file 12711_2021_616_MOESM2_ESM.pdf]

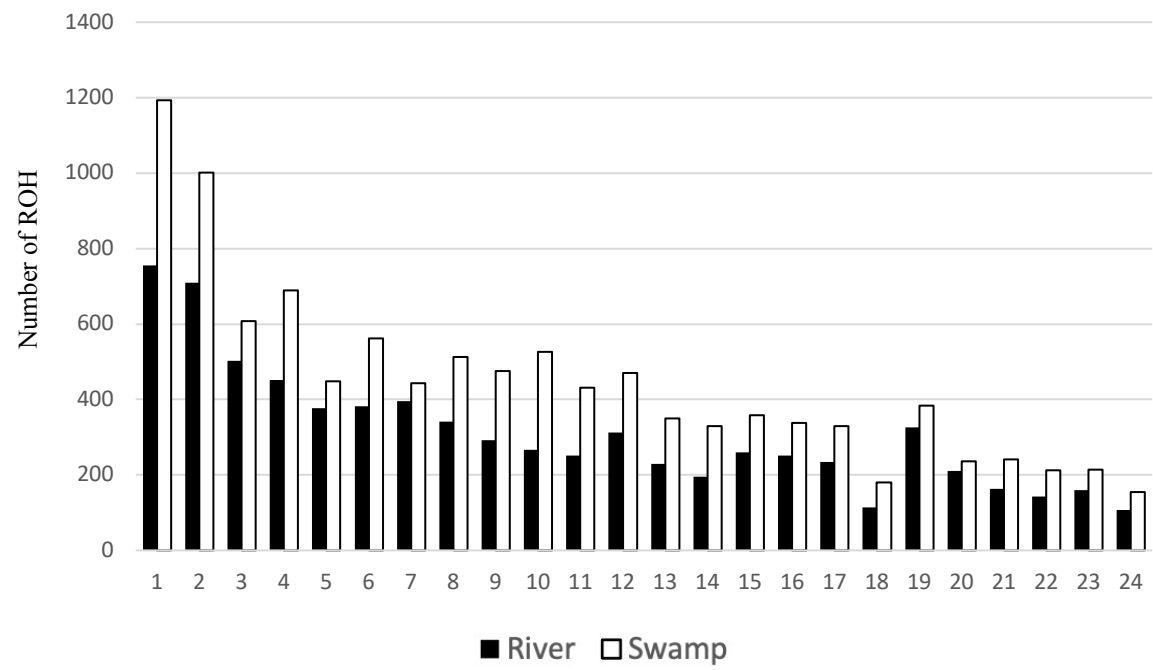

Supplement: Supplementary file 3 — Additional file 3: Figure S2. Frequency distribution of ROH across chromosomes in river (black bars) and swamp (white bars) buffalo. [file 12711_2021_616_MOESM3_ESM.pdf]

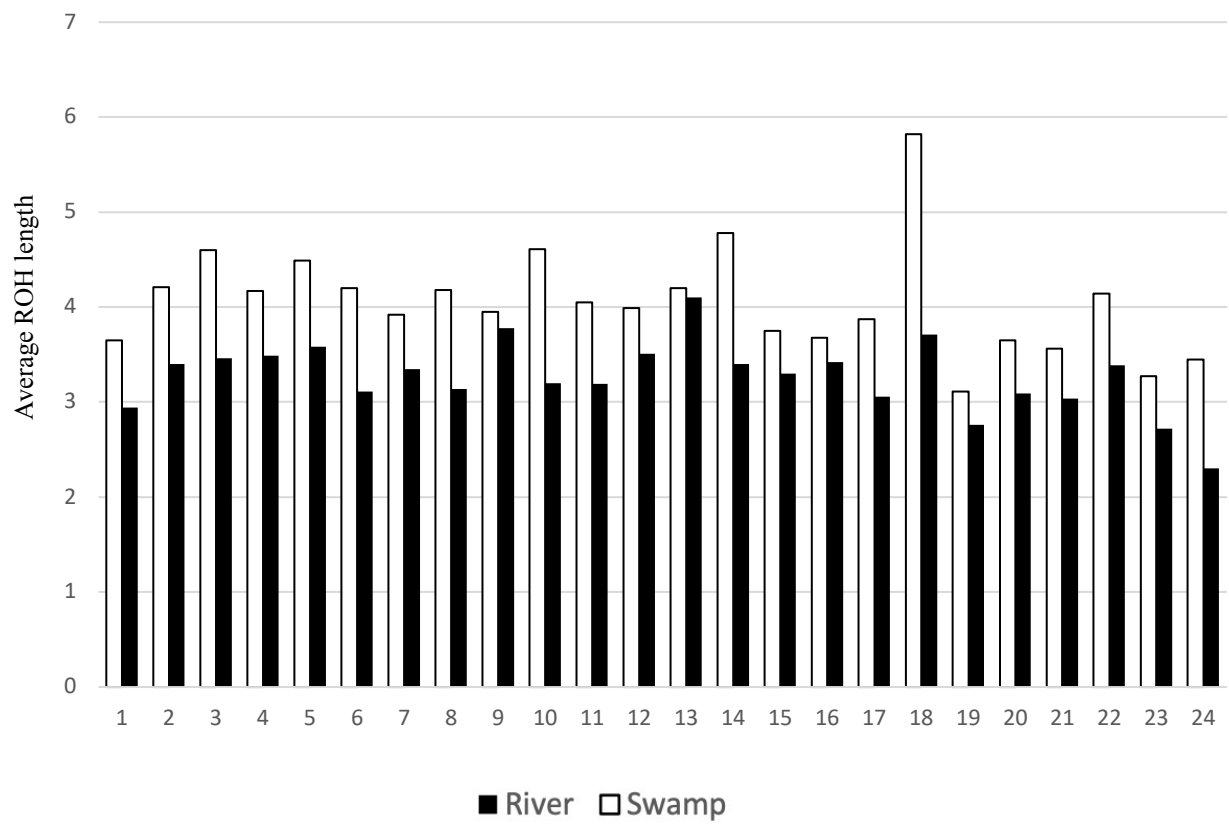

Supplement: Supplementary file 4 — Additional file 4: Figure S3. Distribution of the average ROH length per chromosome in river (white bars) and swamp (black bars) buffalo populations. [file 12711_2021_616_MOESM4_ESM.pdf]

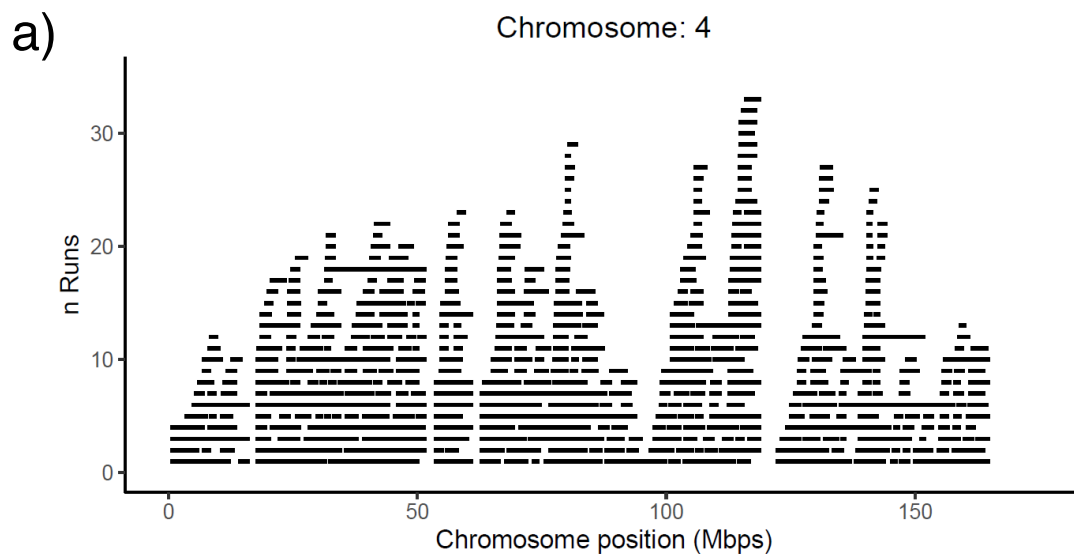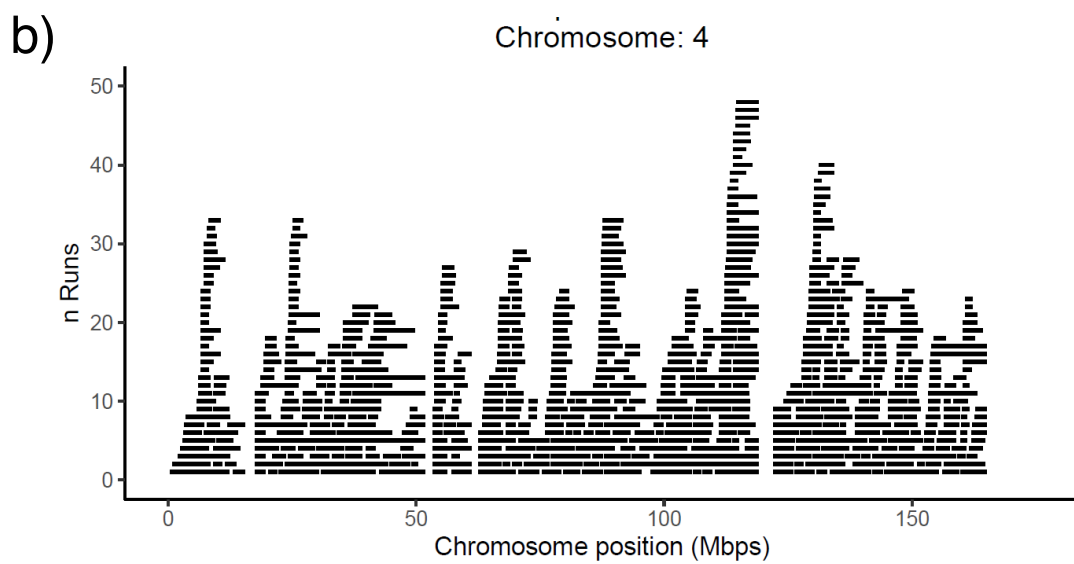

Supplement: Supplementary file 6 — Additional file 6: Figure S4. Stacked bar graph of ROH distribution on BBU4 in river (a) and swamp (b) buffalo. [file 12711_2021_616_MOESM6_ESM.pdf]

a)

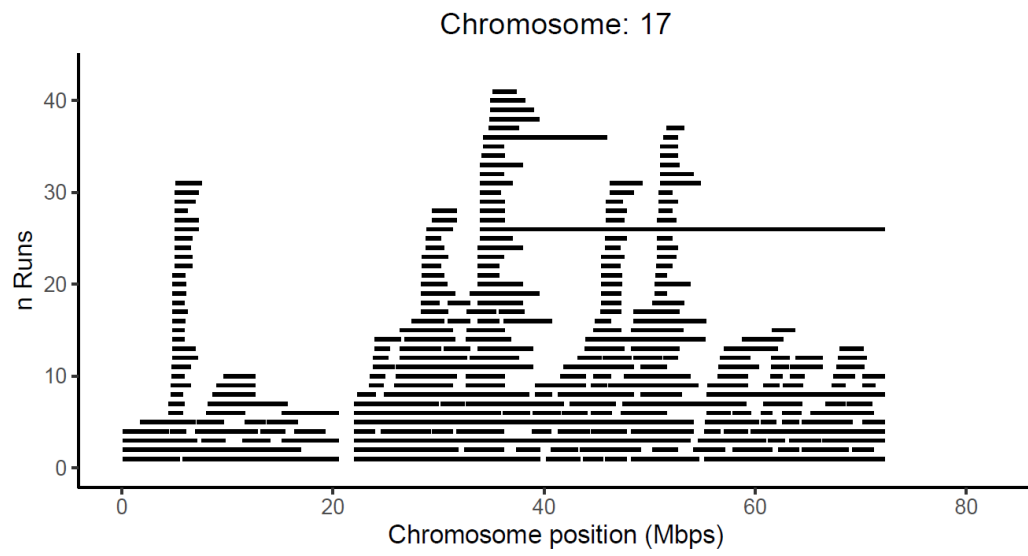

b)

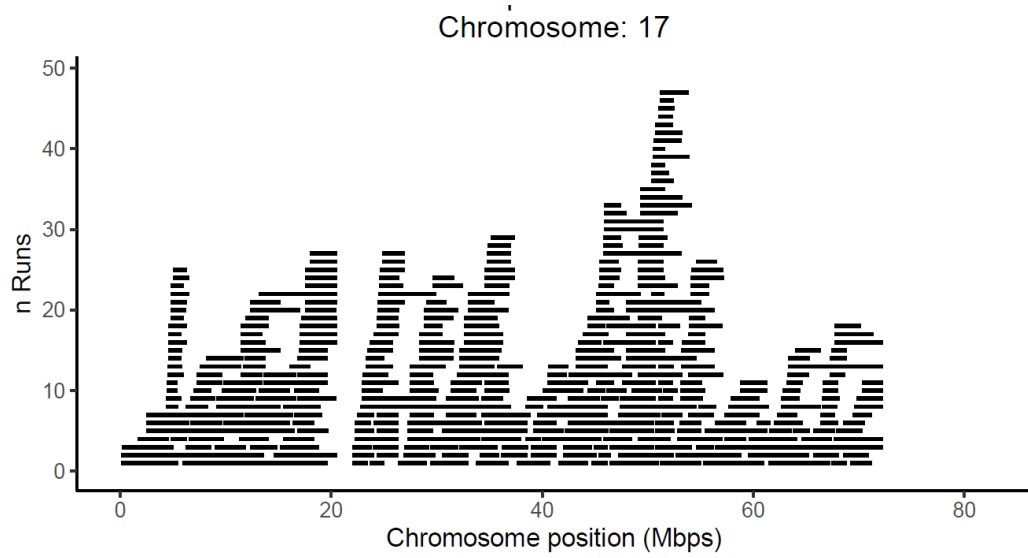

Supplement: Supplementary file 7 — Additional file 7: Figure S5. Stacked bar graph of ROH distribution on BBU17 in river (a) and swamp (b) buffalo. [file 12711_2021_616_MOESM7_ESM.pdf]

CHR 1

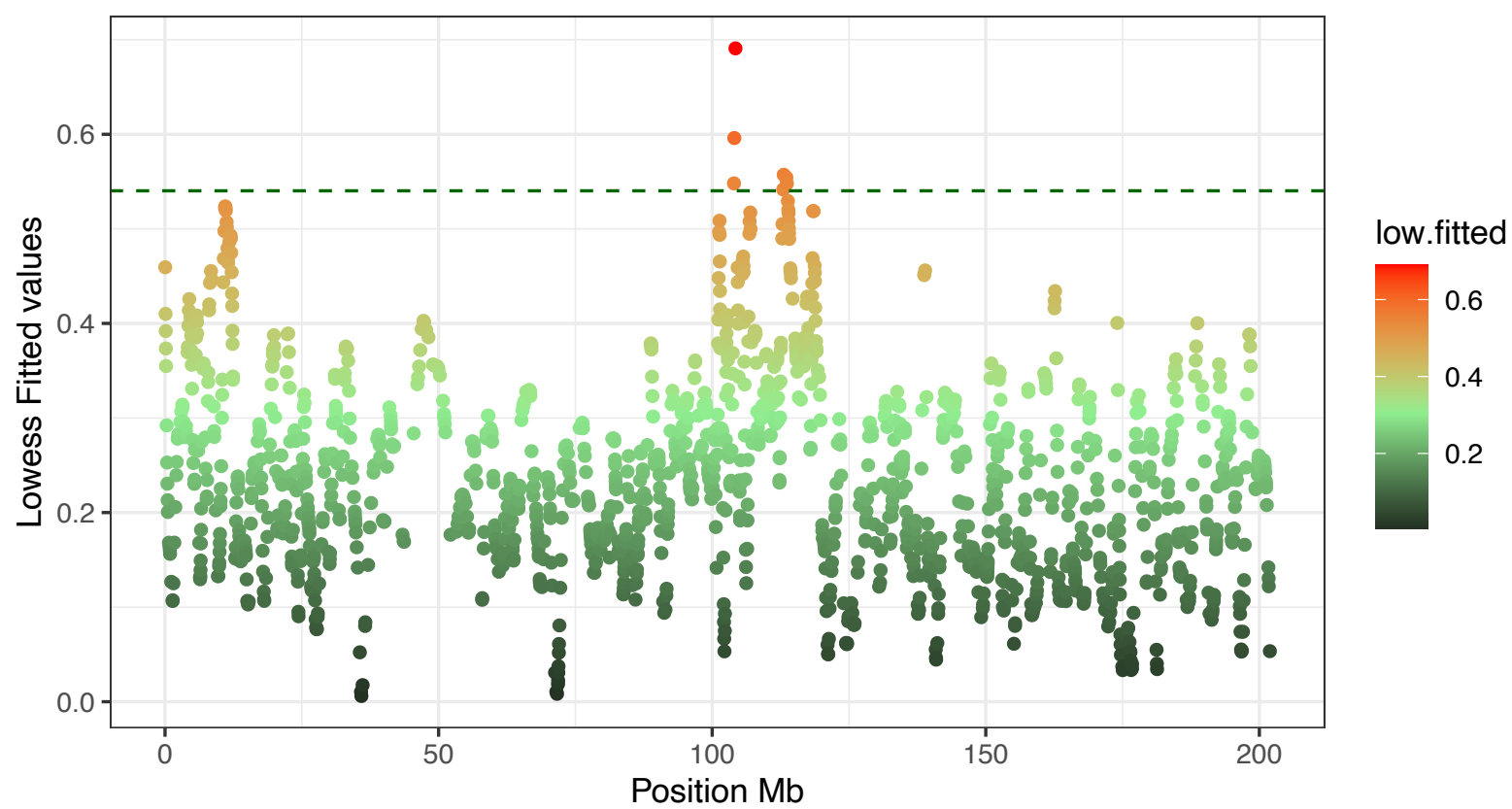

CHR 2

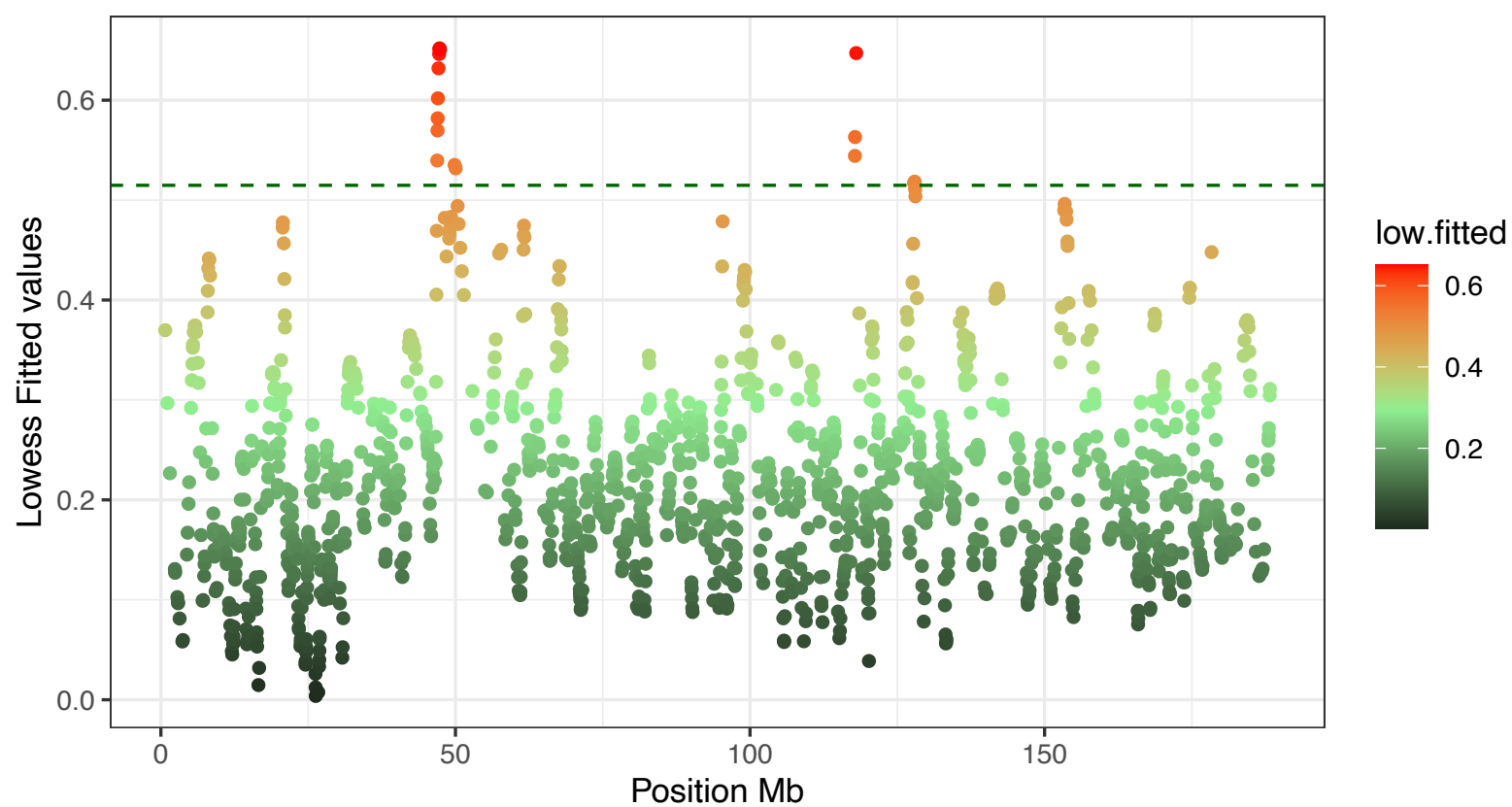

CHR 3

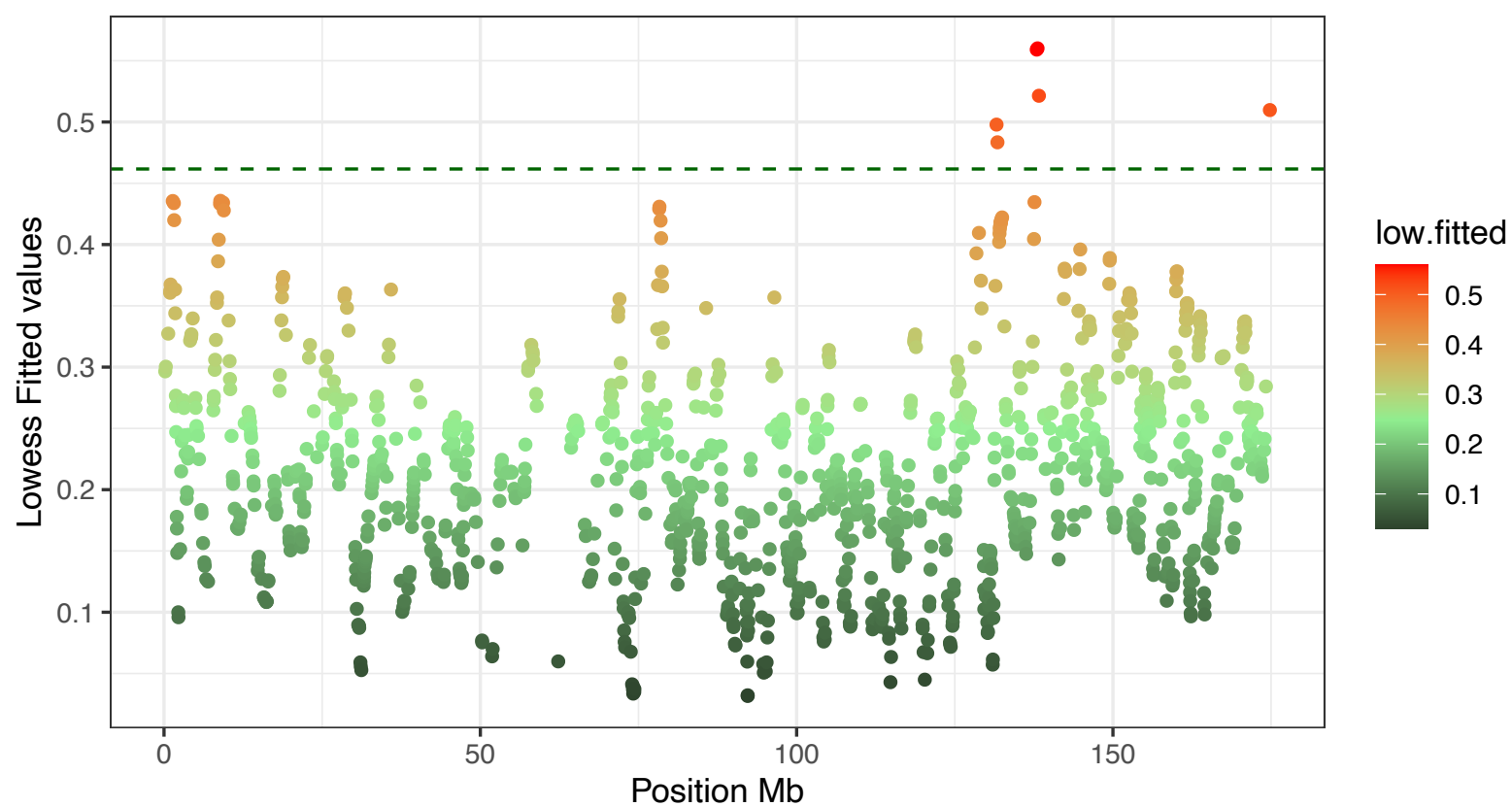

CHR 4

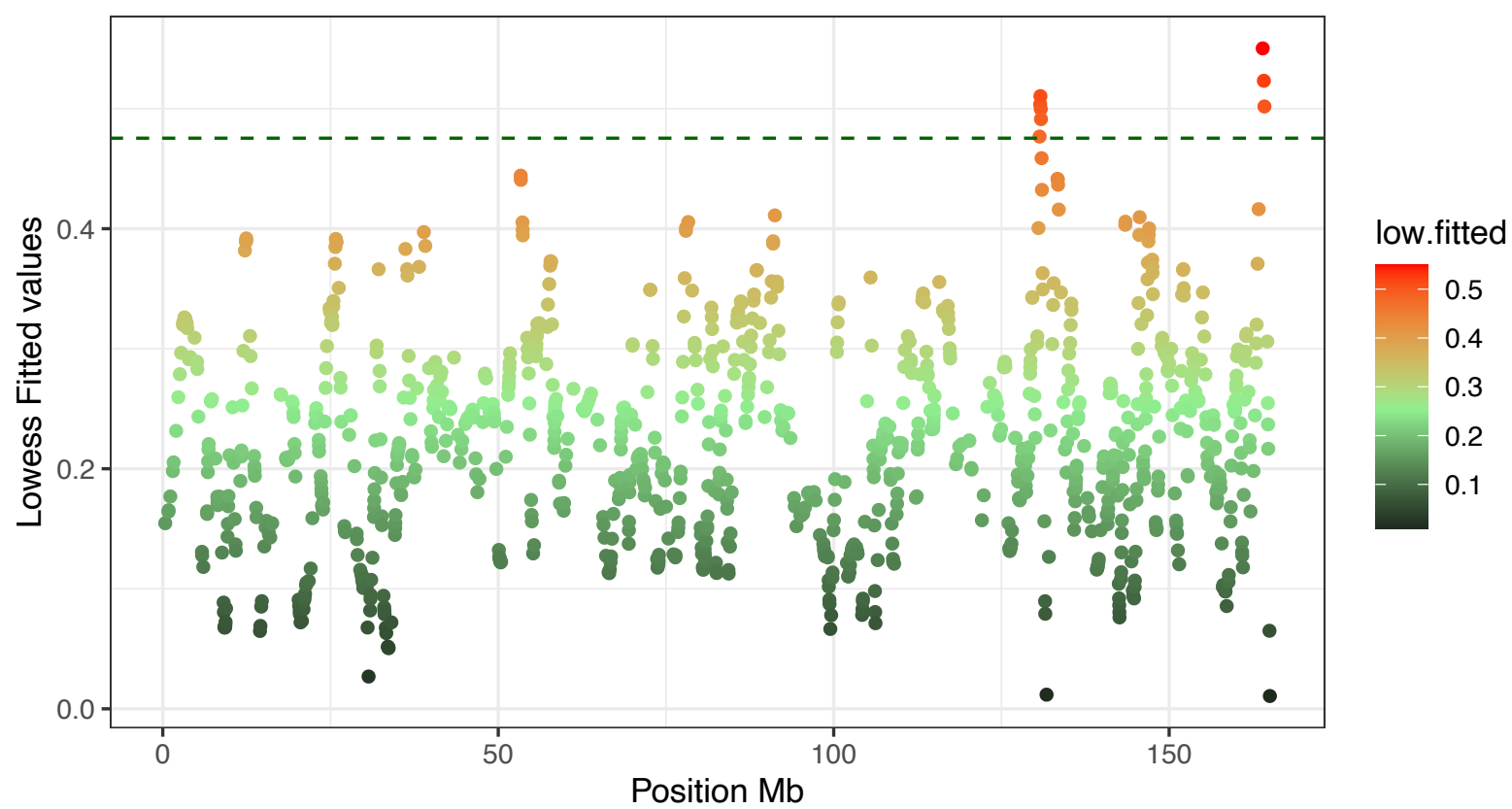

CHR 5

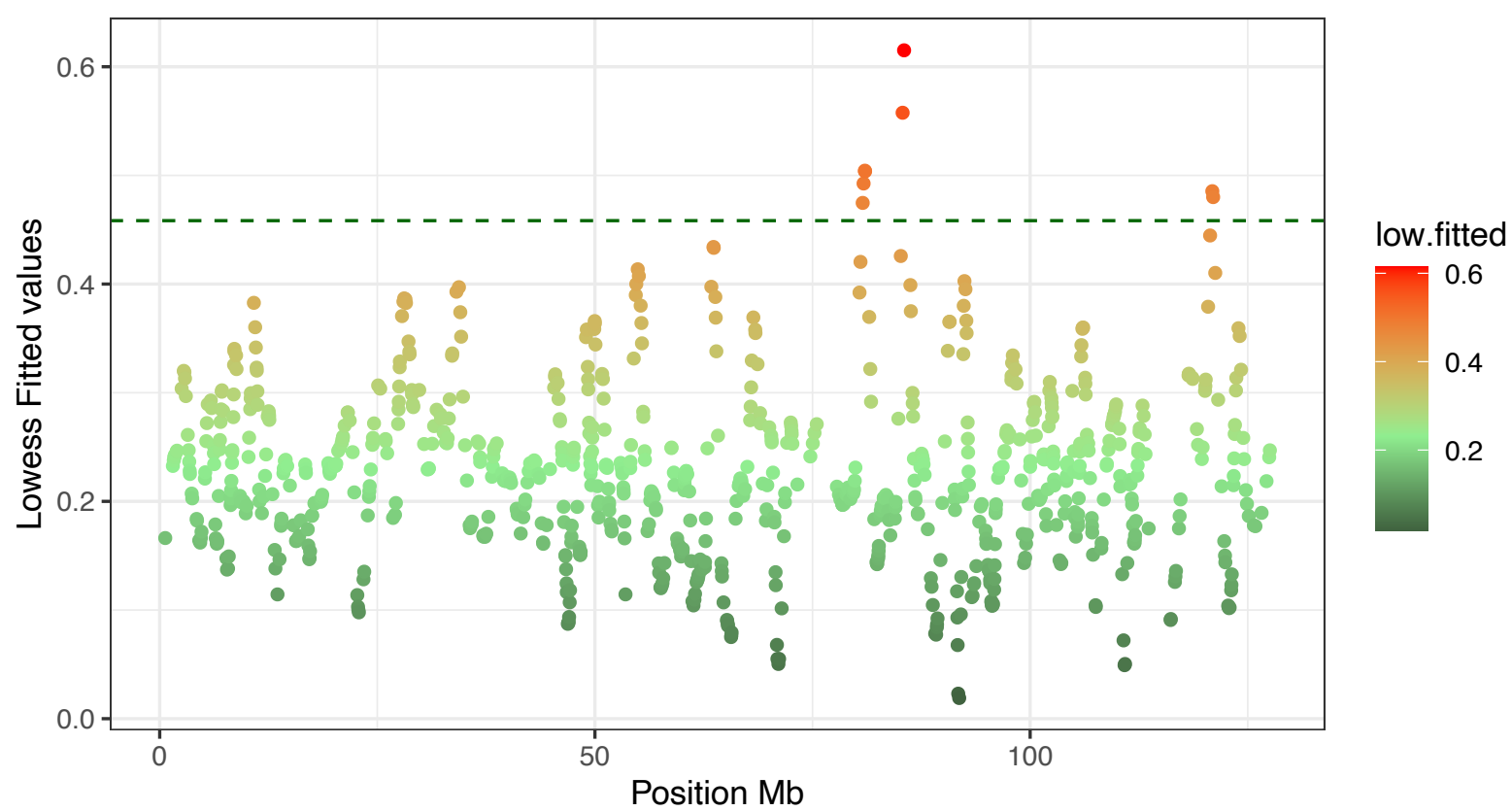

CHR 6

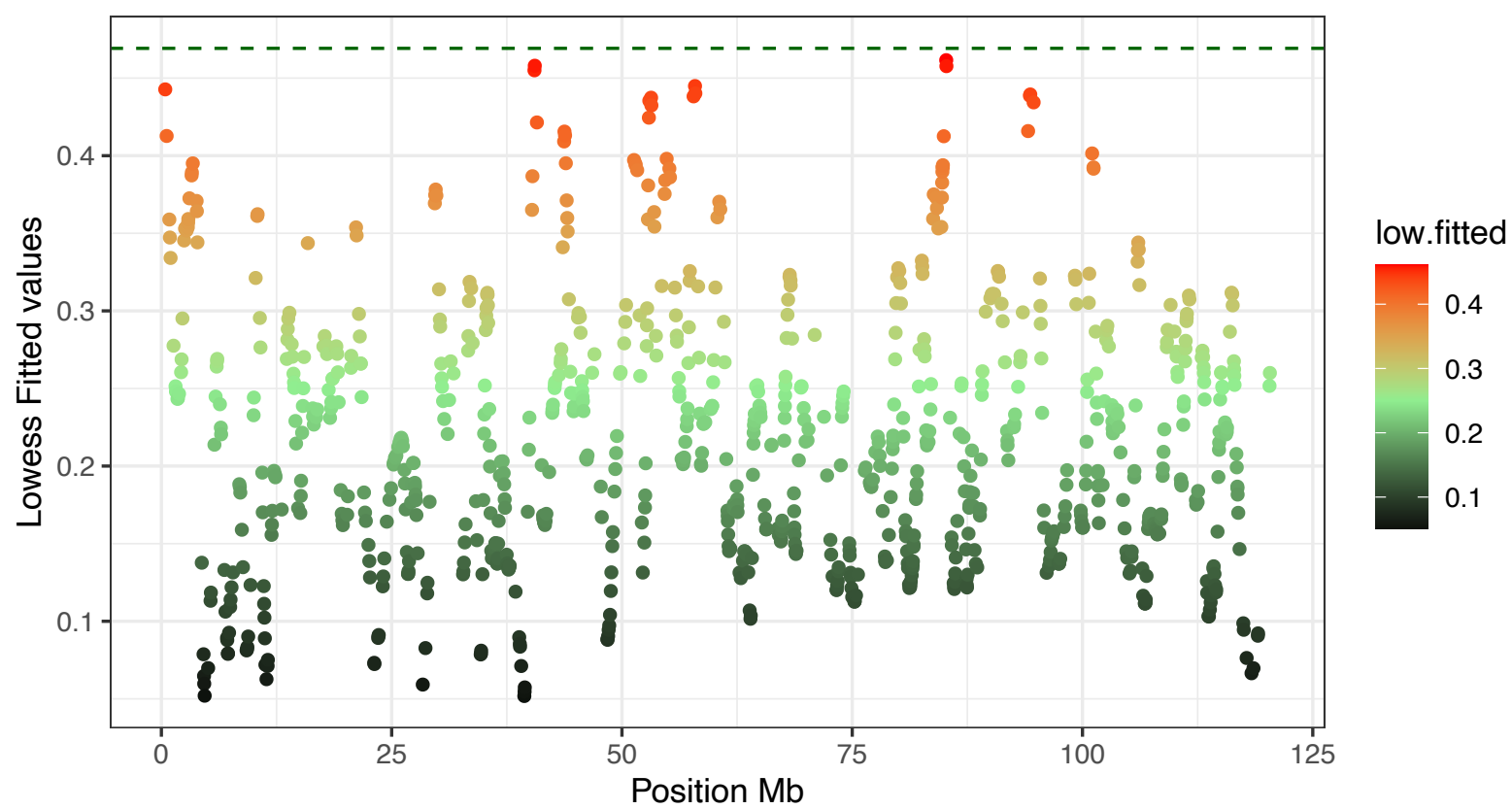

CHR 7

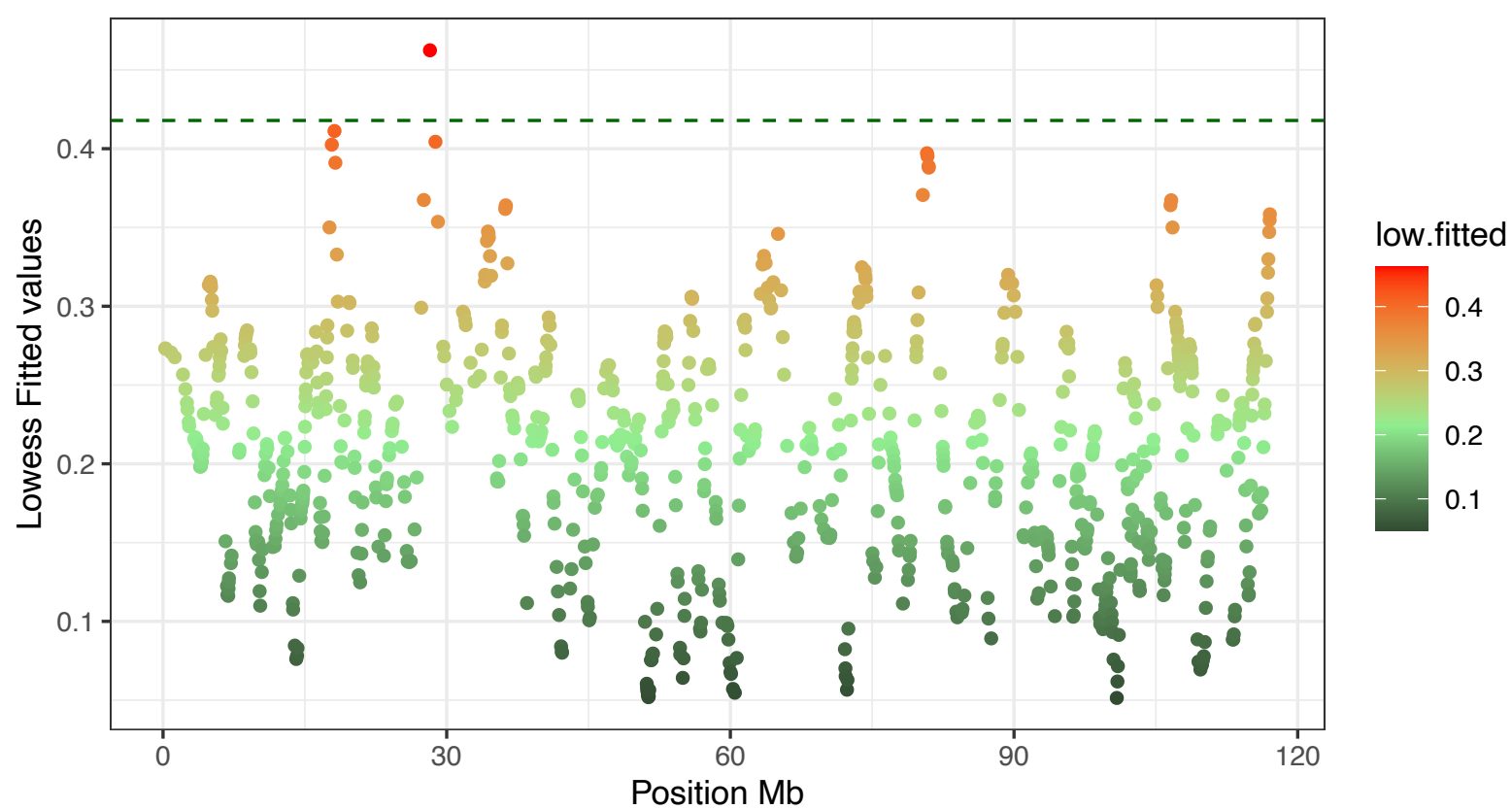

CHR 8

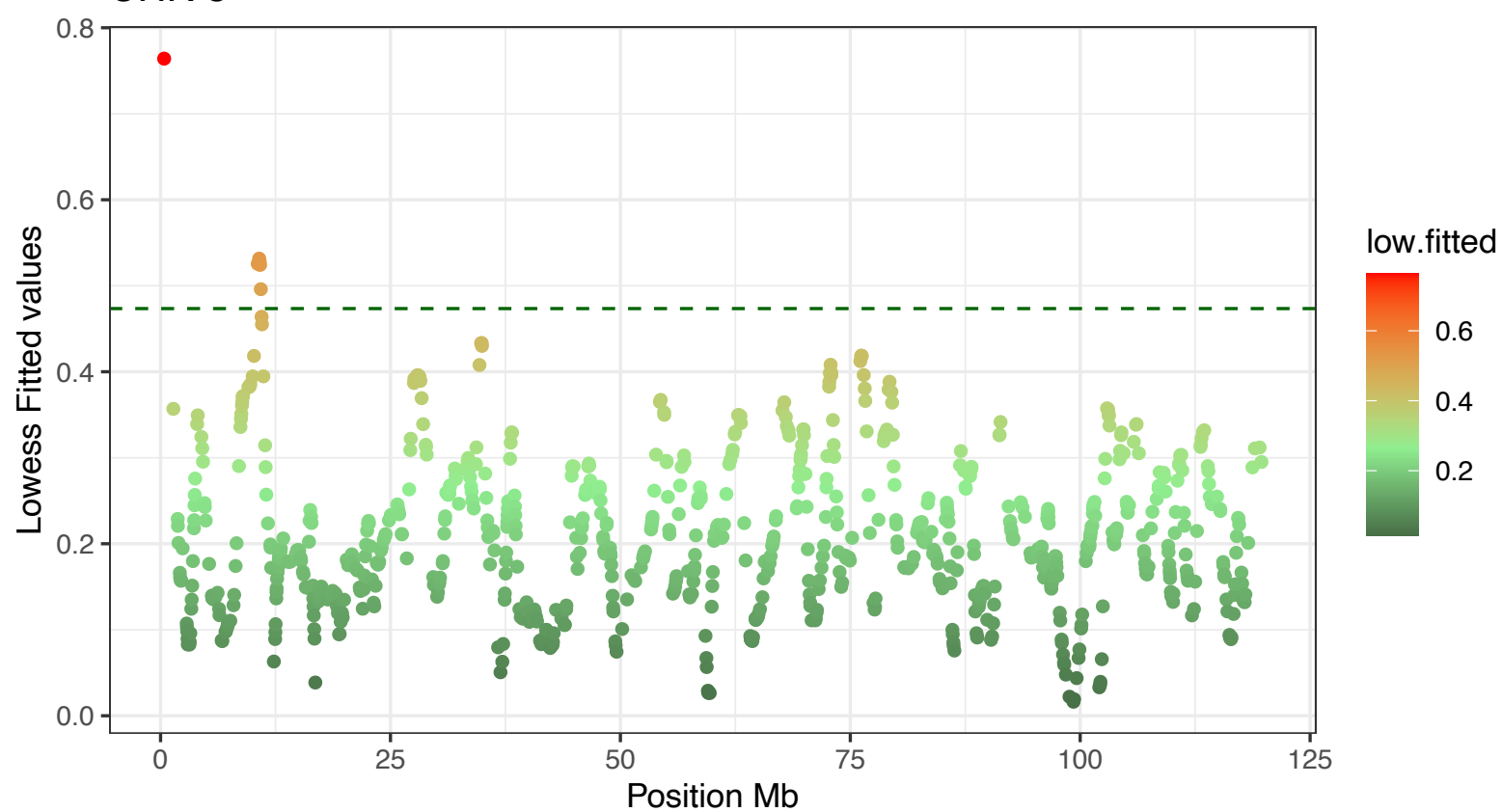

CHR 9

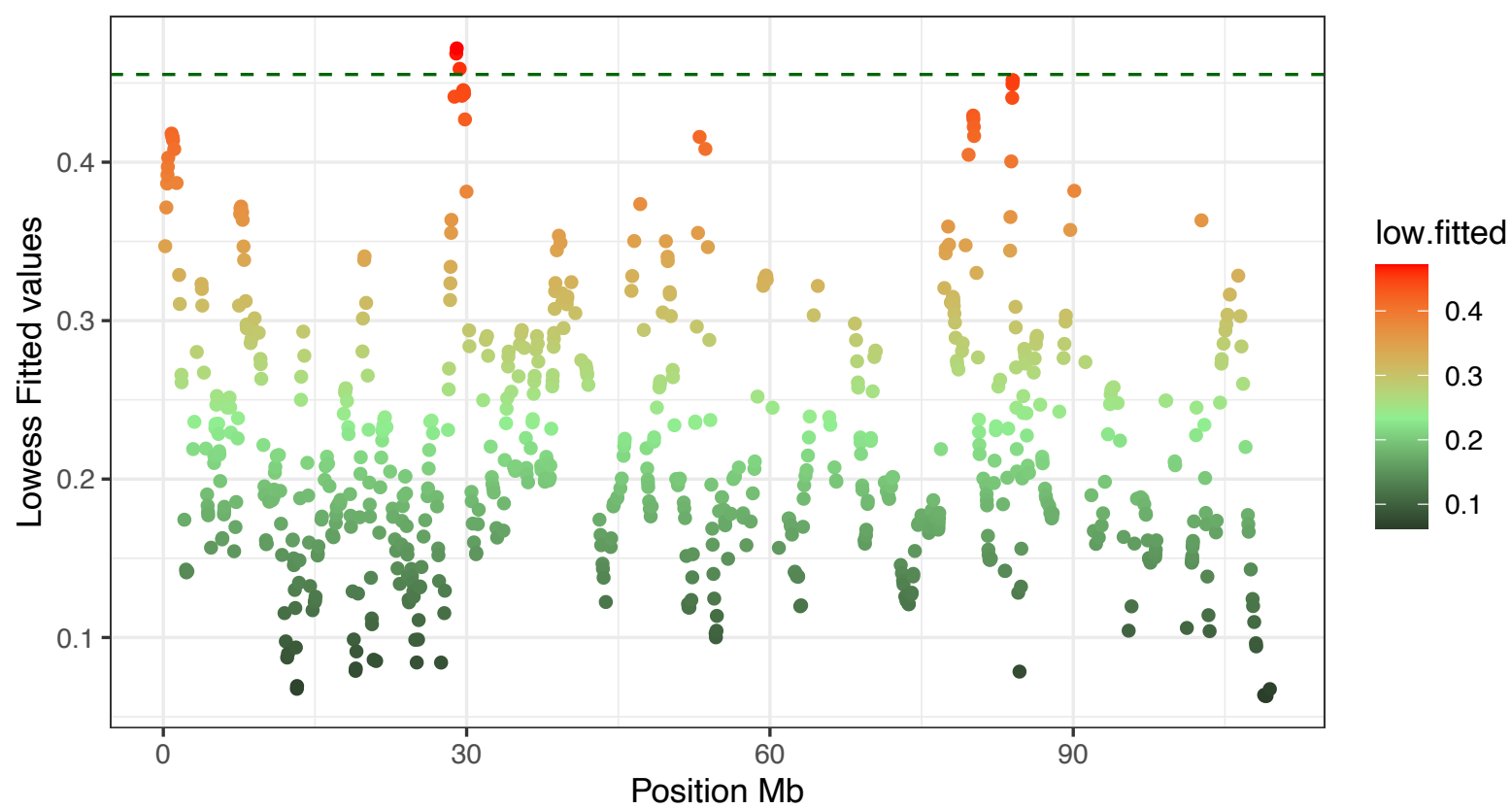

CHR 10

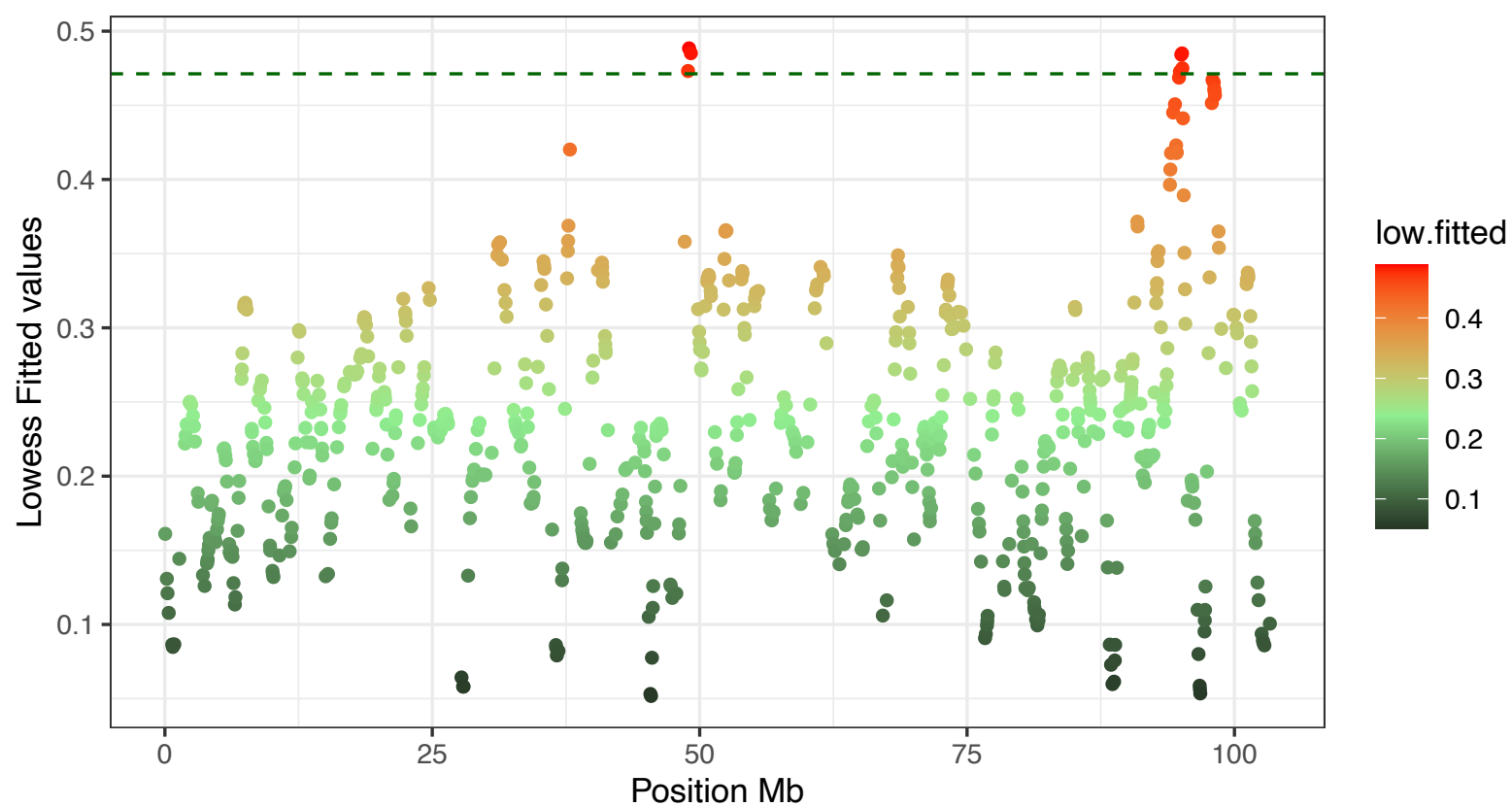

CHR 11

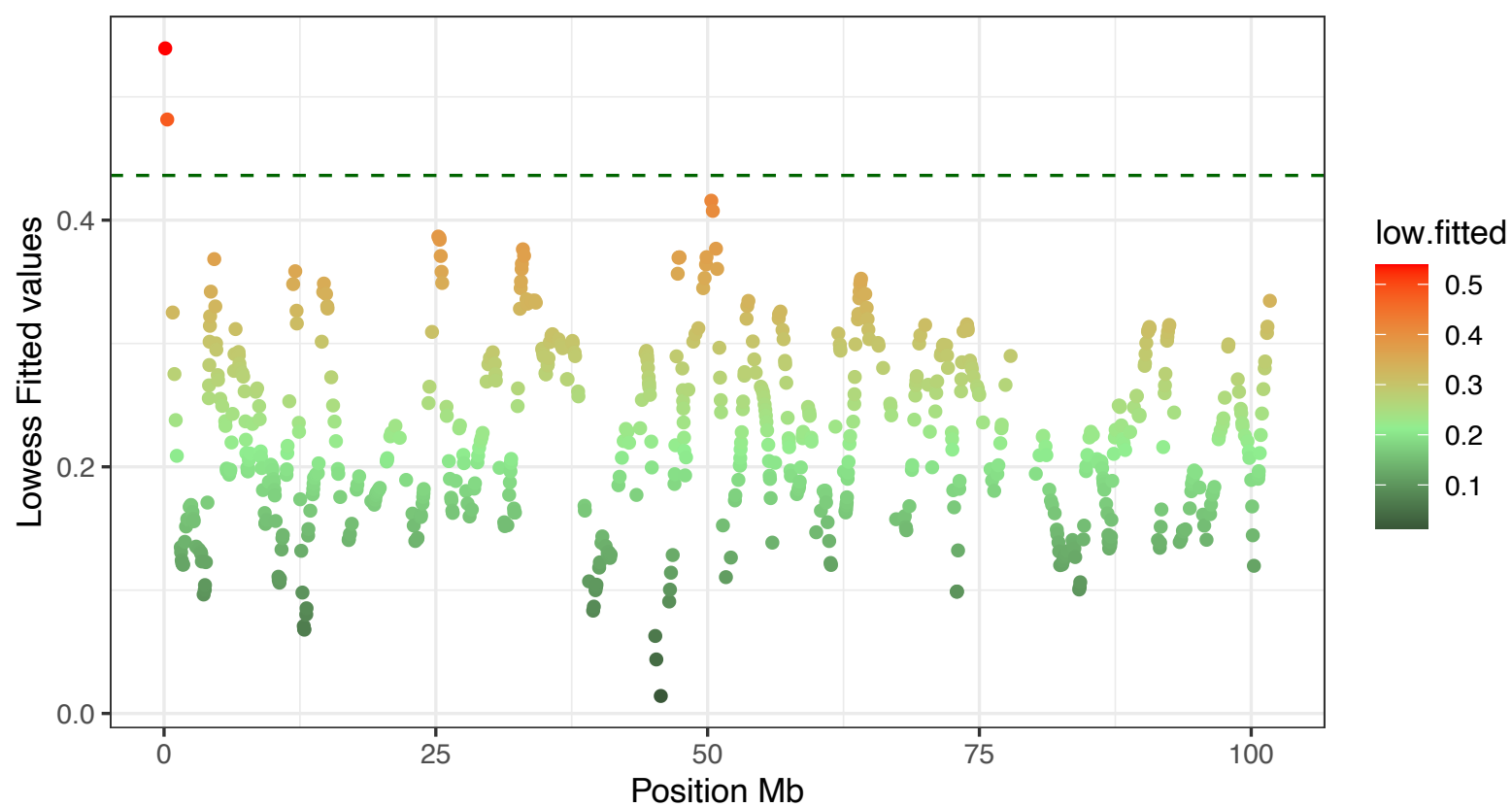

CHR 12

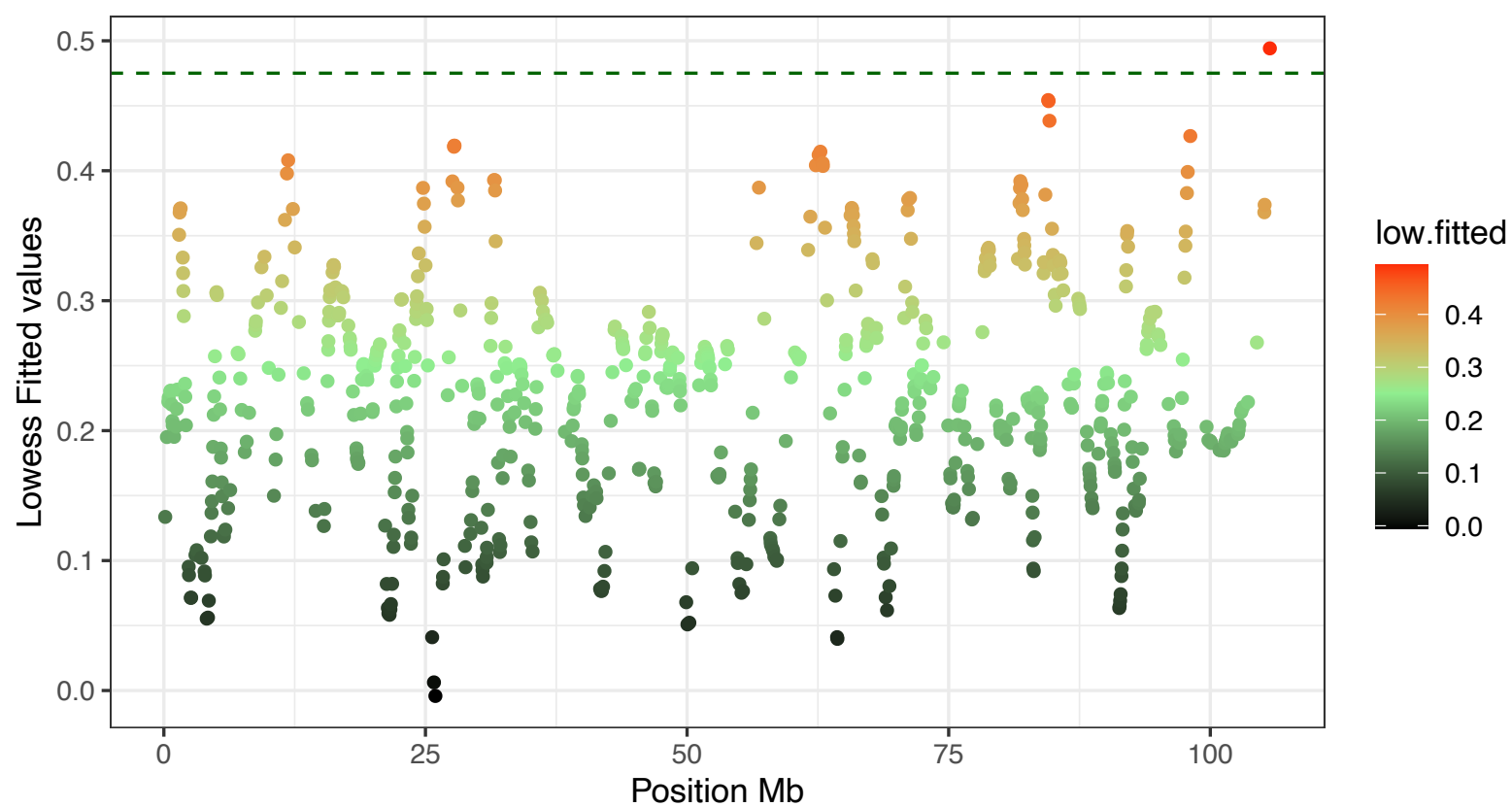

CHR 13

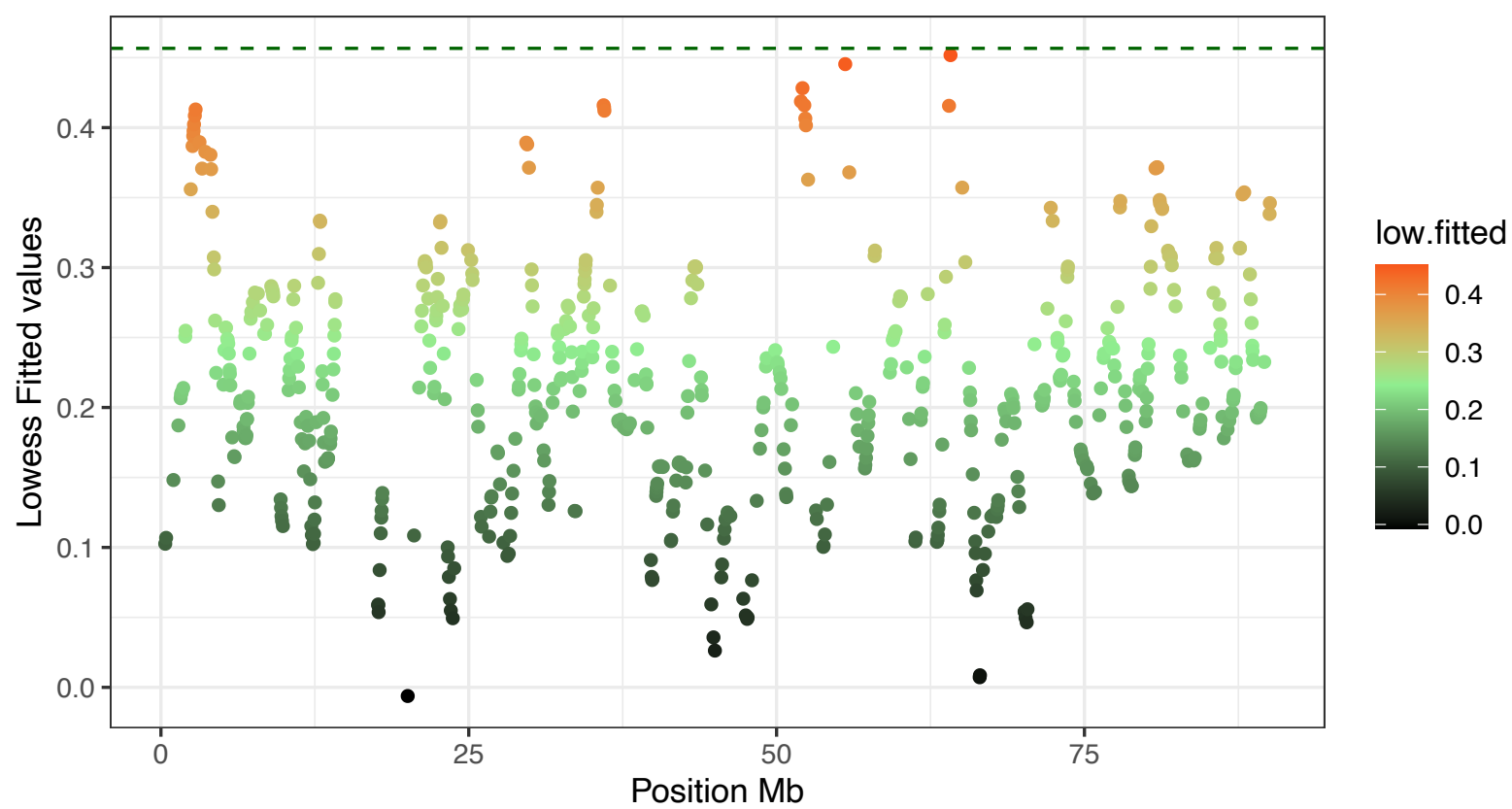

CHR 14

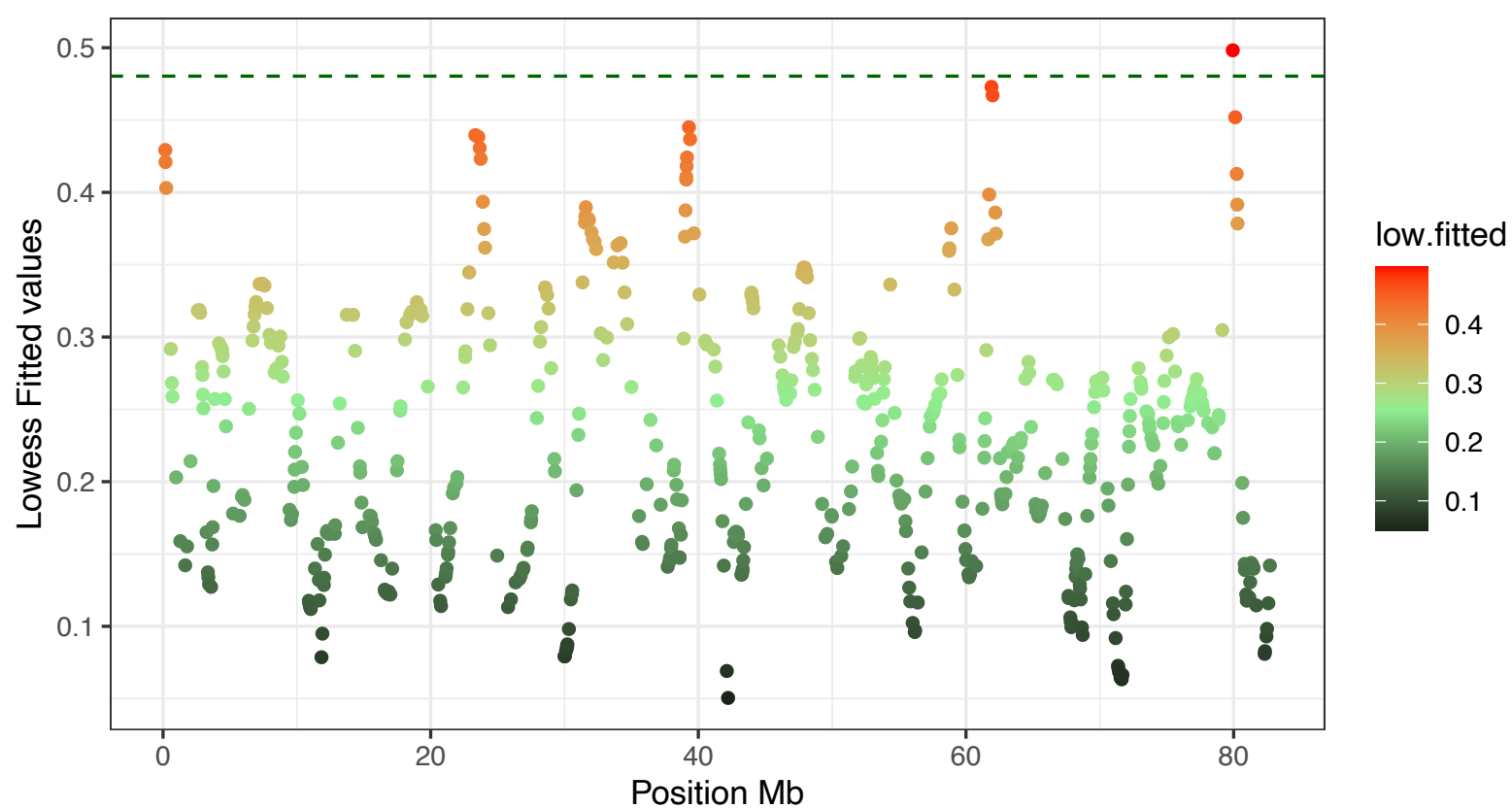

CHR 15

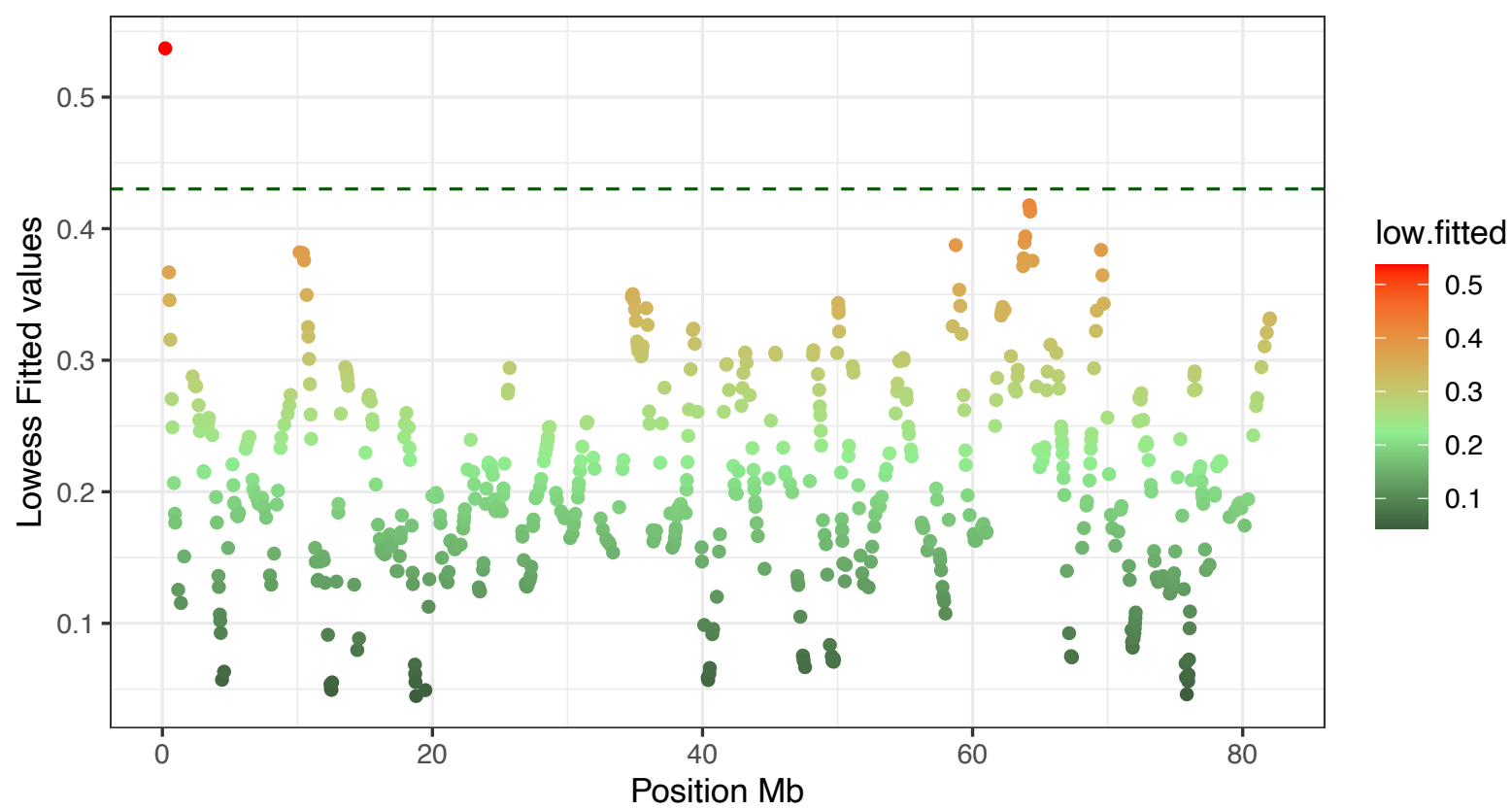

CHR 16

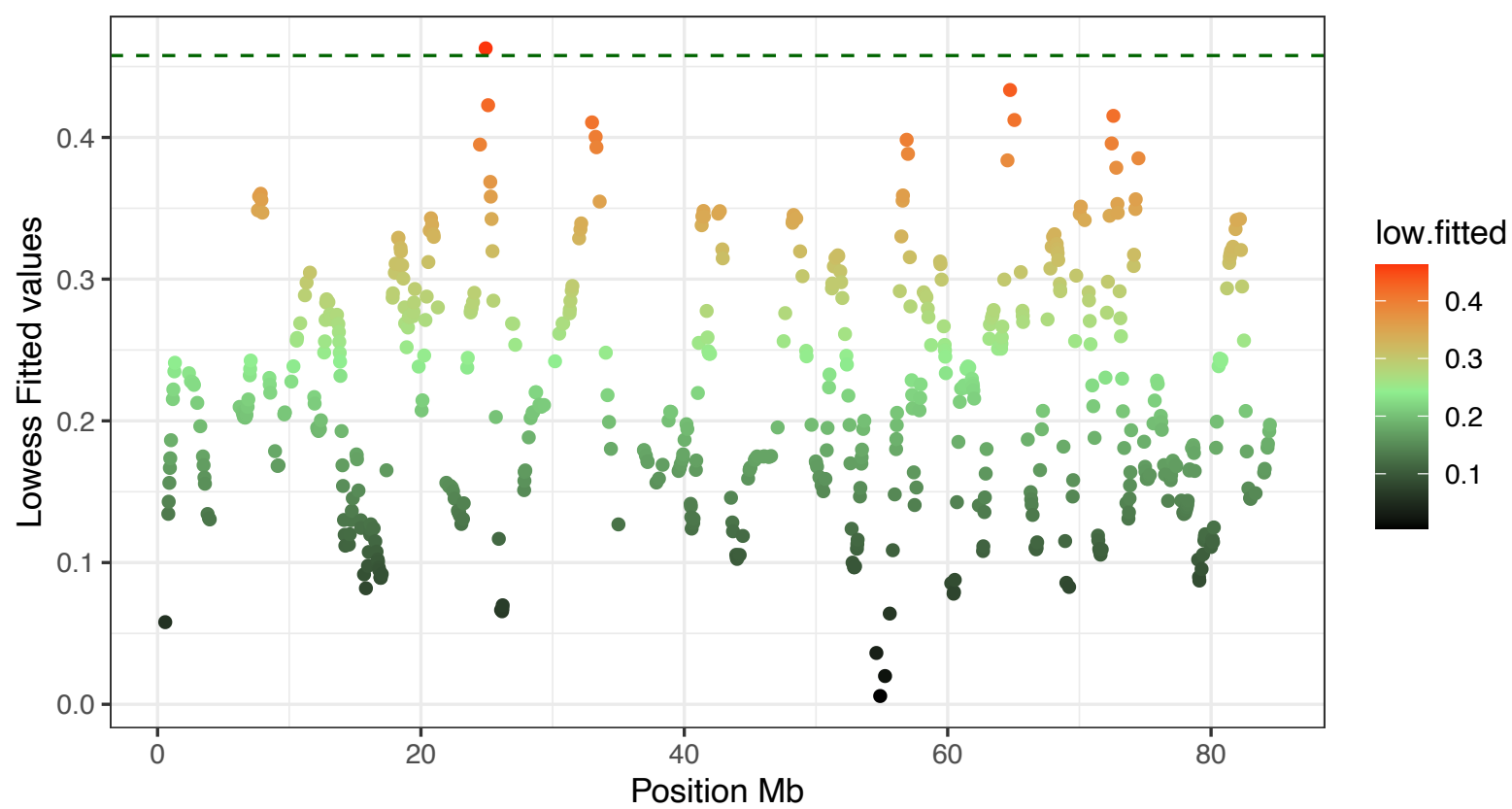

CHR 17

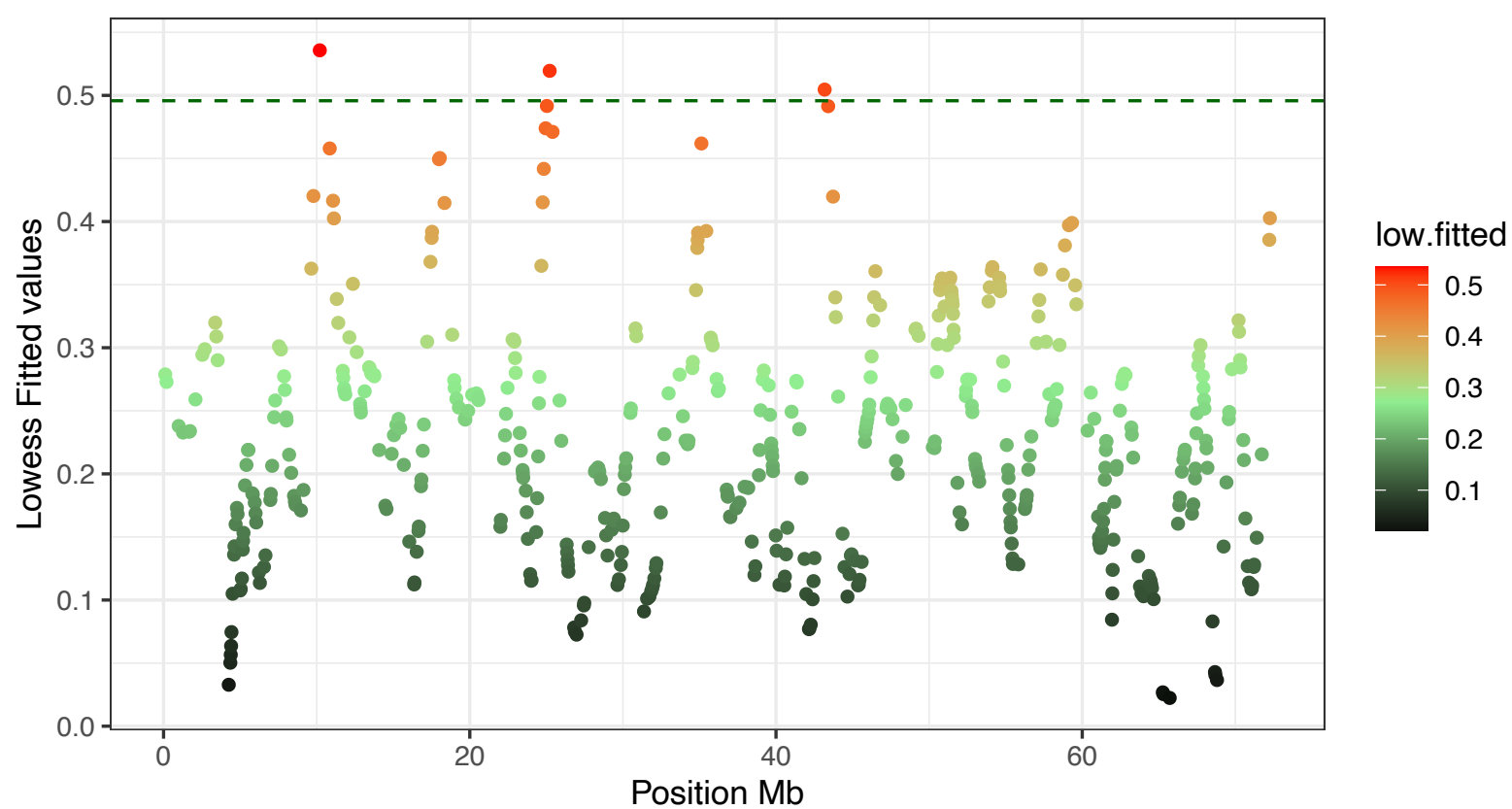

CHR 18

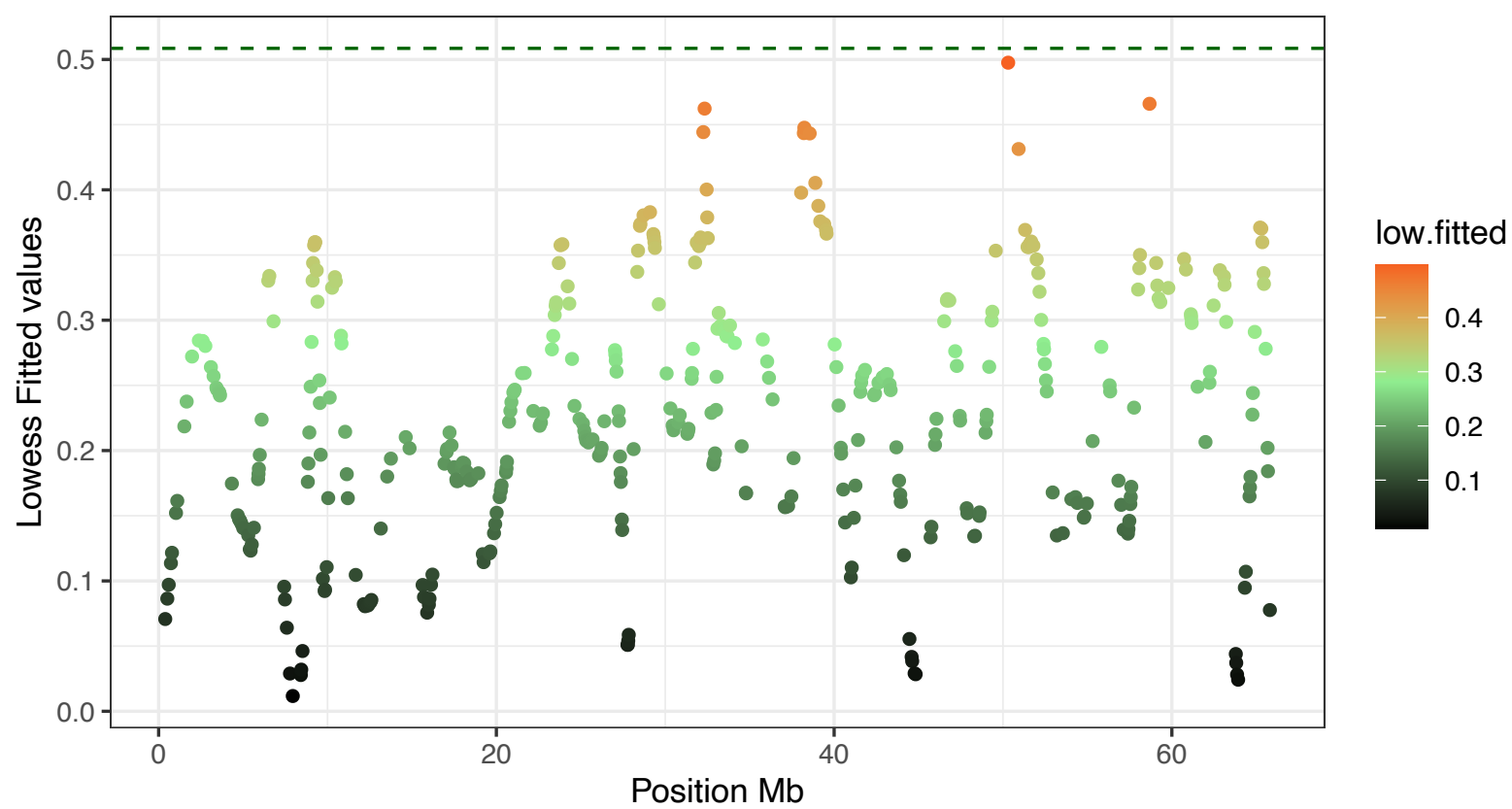

CHR 19

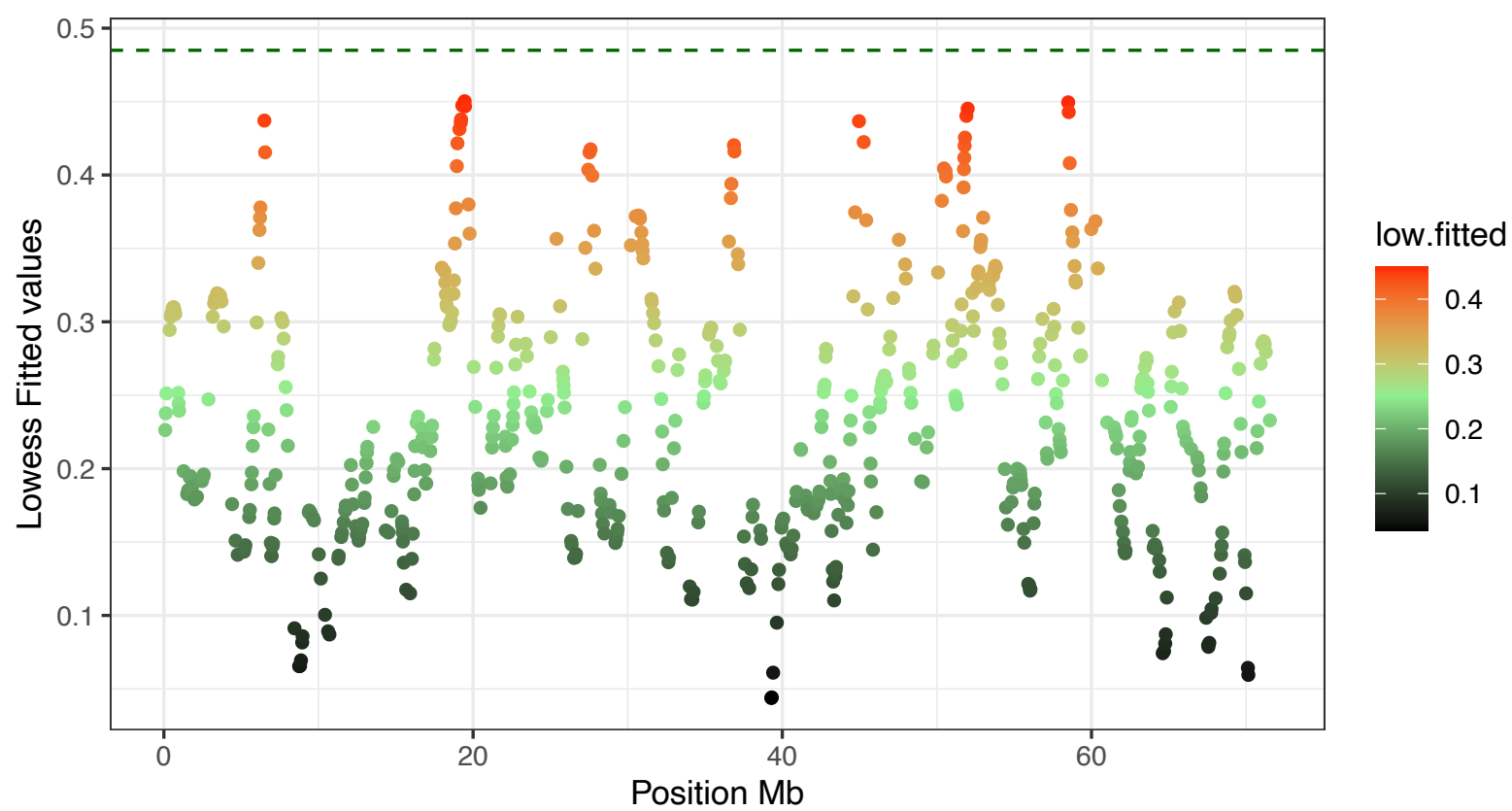

CHR 20

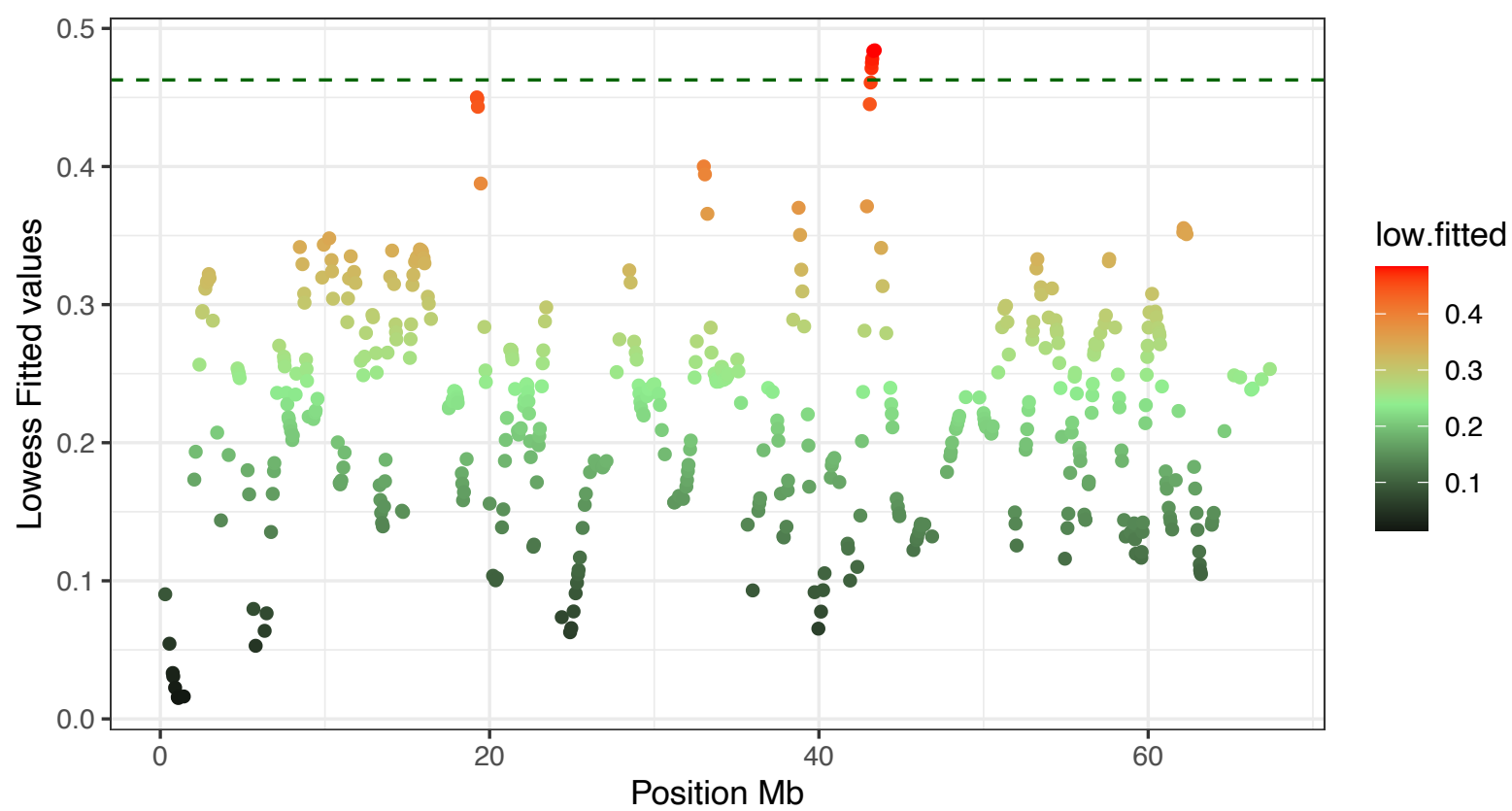

CHR 21

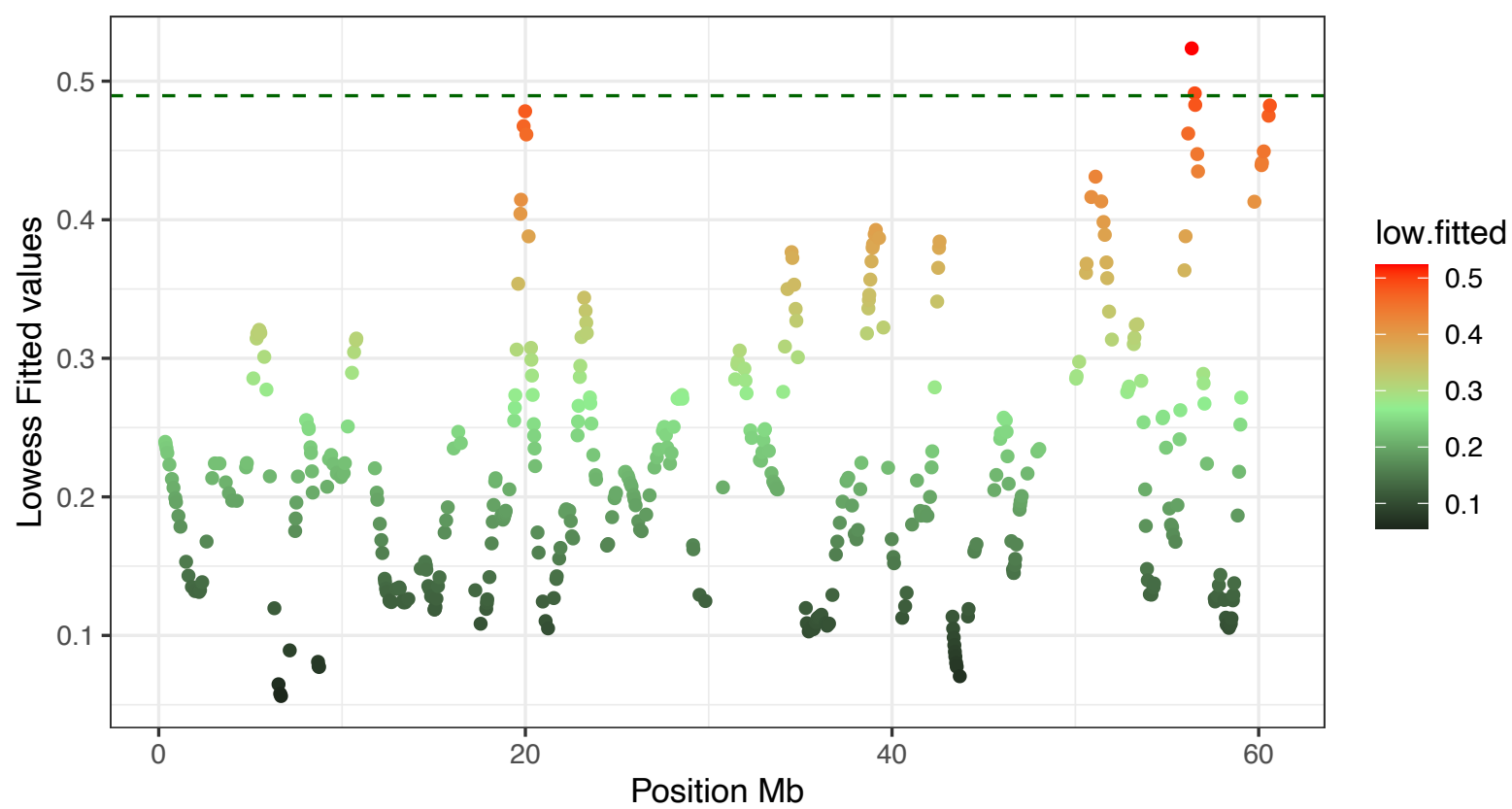

CHR 22

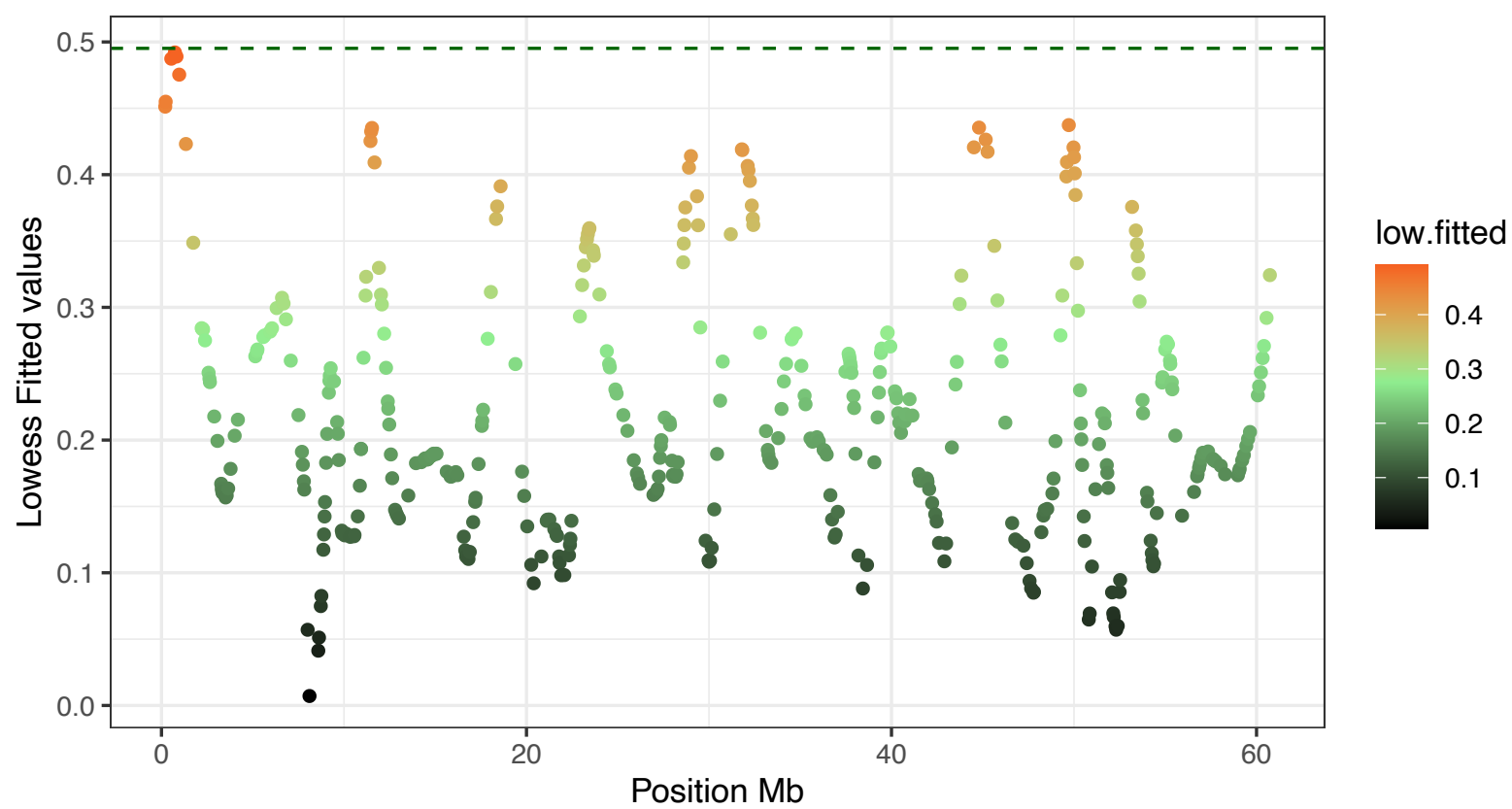

CHR 23

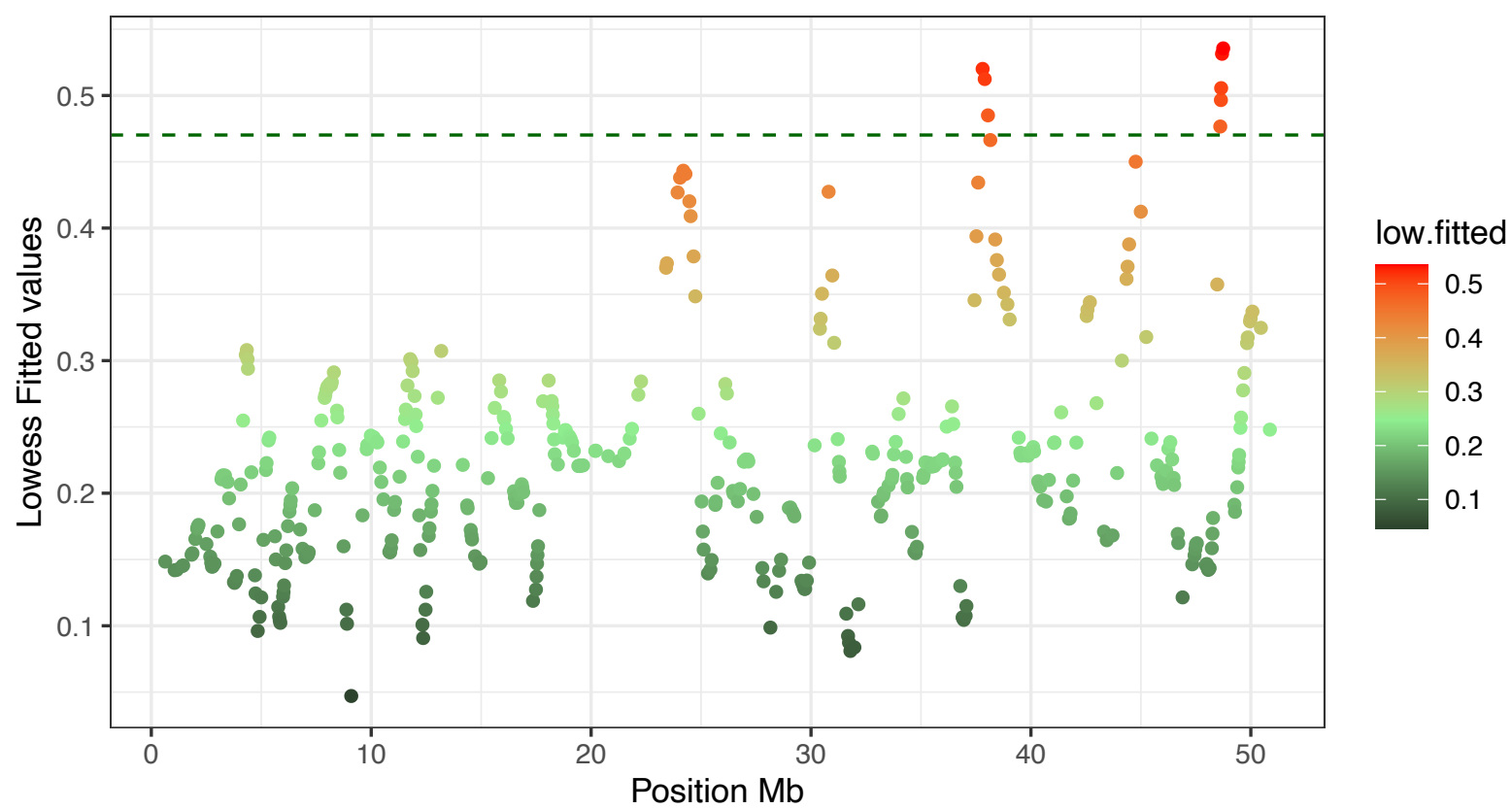

CHR 24

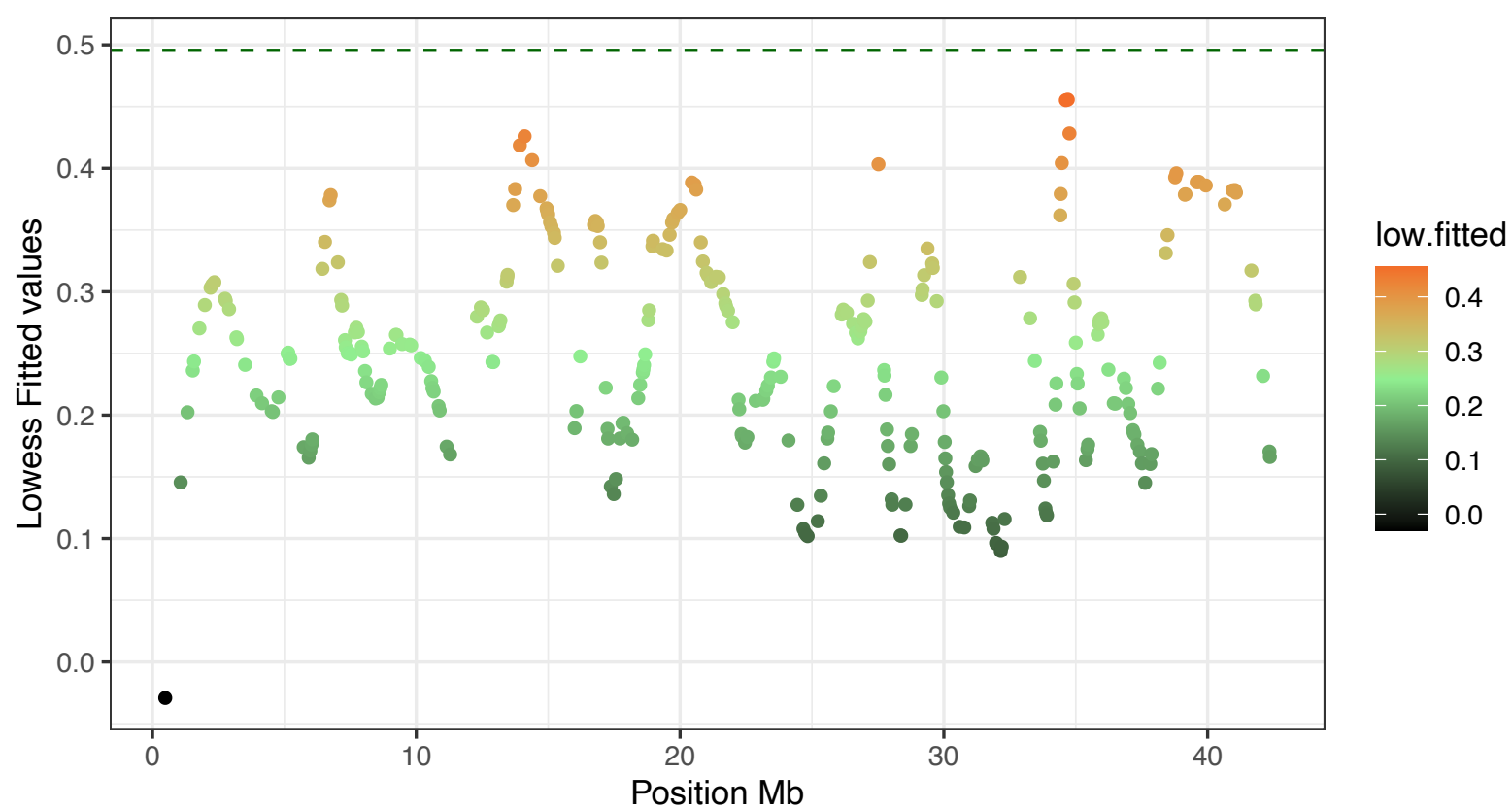

Supplement: Supplementary file 8 — Additional file 8: Figure S6. Manhattan plot of smoothed FST values for chromosomes 1 to 24. [file 12711_2021_616_MOESM8_ESM.pdf]

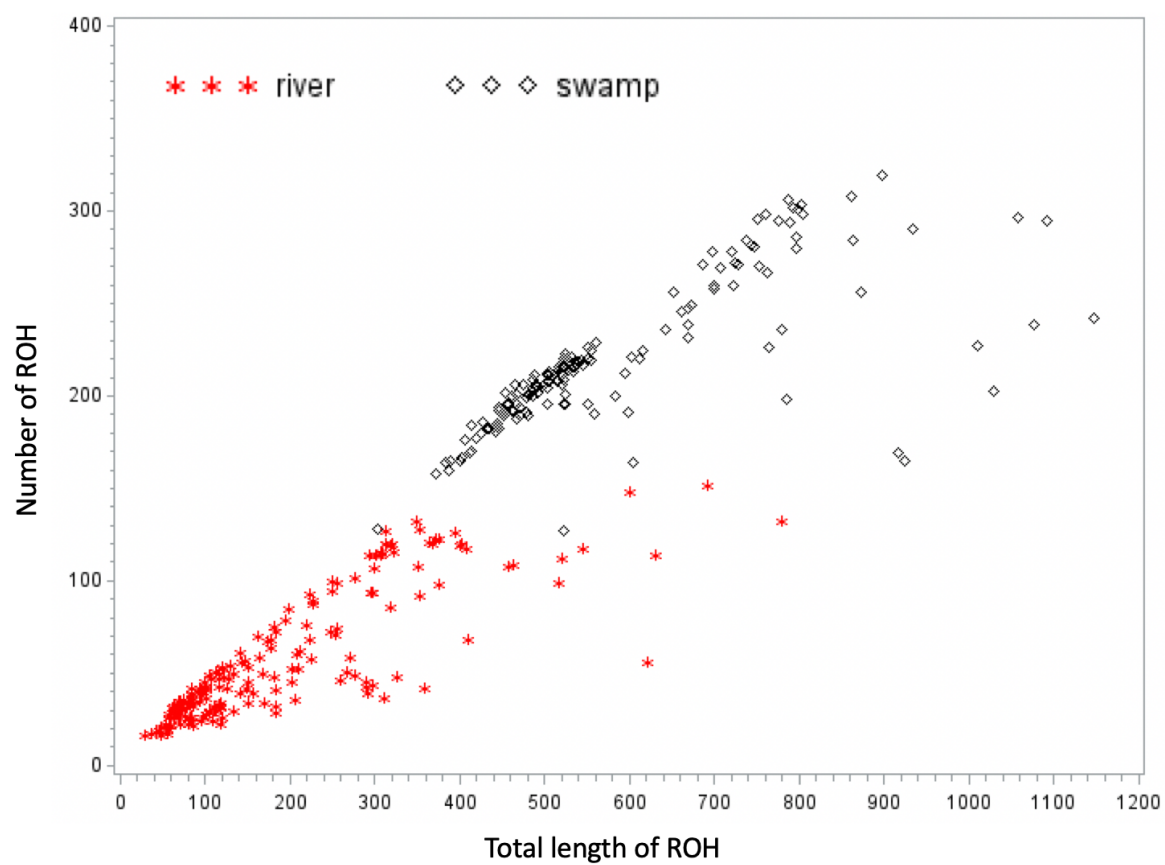

Supplement: Supplementary file 12 — Additional file 12: Figure S7. Relationship between number of ROH and total length of the genome covered by ROH (using all ROH detected in ALL_DATA set). [file 12711_2021_616_MOESM12_ESM.pdf]

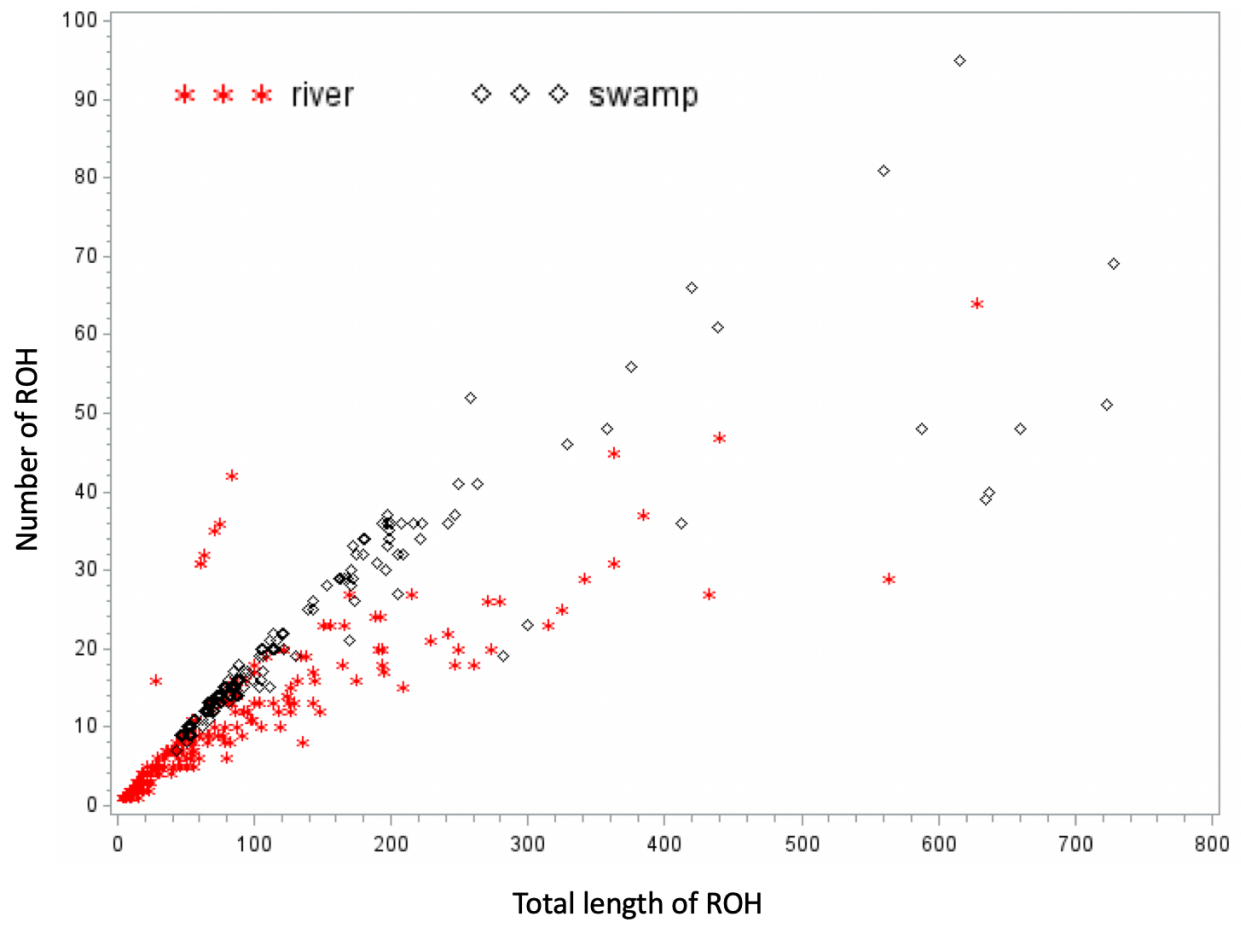

Supplement: Supplementary file 13 — Additional file 13: Figure S8. Relationship between number of ROH and total length of the genome covered by ROH (using ROH with length ≥ 4 Mb detected in the ALL_DATA set). [file 12711_2021_616_MOESM13_ESM.pdf]
