# Supplementary material for: The distribution of runs of homozygosity in the genome of river and swamp buffaloes reveals a history of adaptation, migration and crossbred events
Source: Genet Sel Evol. 2021 Feb 27;53:20. doi: 10.1186/s12711-021-00616-3 (PMC7912491; doi:10.1186/s12711-021-00616-3)
Supplement: Supplementary file 5 — Additional file 5: Table S2. Top ten most frequently detected ROH in the ALL_DATA set. [file 12711_2021_616_MOESM5_ESM.docx]

| Chr. | Start | Length | n. | Breed/population |
| --- | --- | --- | --- | --- |
| 16 | 81.6 Mb | 2.8 Mb | 9 | RIVIR_AZA, RIV_MED, RIVMZ, SWACN_YAN, SWAIN_JAV, SWAPH |
| 1 | 11.0 Mb | 1.3 Mb | 7 | SWACN_HUN, SWACN_YAN, SWAIN_JAV, SWAIN_NUT |
| 2 | 50.8 Mb | 7.6 Mb | 7 | SWACN_FUL, SWACN_GUI, SWACN_YAN, SWACN_YIB, SWAIN_JAV, SWAIN_SUM |
| 23 | 28.4 Mb | 1.7 Mb | 6 | RIV_MED, RIVMZ, |
| 2 | 46.2 Mb | 2.7 Mb | 6 | SWACN_FUL, SWACN_GUI, SWACN_HUN, SWAPH |
| 2 | 49.1 Mb | 7.1 Mb | 6 | SWACN_FUL, SWACN_HUN, SWACN_YIB, SWAPH |
| 10 | 68.0 Mb | 1.7 Mb | 6 | SWACN_ENS, SWACN_FUL, SWACN_GUI, SWAIN_JAV |
| 16 | 73.0 Mb | 1.4 Mb | 6 | RIV_MED, RIVMZ |
| 10 | 101.1 Mb | 2.2 Mb | 6 | RIVCO, SWAIN_NUT, SWAIN_SUW, SWAPH |
| 1 | 118.1 Mb | 1.2 Mb | 6 | SWACN_ENS, SWACN_YAN, SWAIN_JAV, SWAIN_NUT, SWAPH |
