# Supplementary material for: The distribution of runs of homozygosity in the genome of river and swamp buffaloes reveals a history of adaptation, migration and crossbred events
Source: Genet Sel Evol. 2021 Feb 27;53:20. doi: 10.1186/s12711-021-00616-3 (PMC7912491; doi:10.1186/s12711-021-00616-3)
Supplement: Supplementary file 9 — Additional file 9: Table S3. Top five most frequently detected ROH in RIVER_DATA and SWAMP_DATA sets. [file 12711_2021_616_MOESM9_ESM.docx]

| **RIVER_DATA** | | | | |
| --- | --- | --- | --- | --- |
| Chr. | Start (Mb) | Length (Mb) | n. | Breed/population |
| 1 | 42.85 | 1.15 | 9 | RIVIT_MED, RIVMZ |
| 3 | 60.18 | 3.54 | 9 | RIVBR_MUR, RIVIR_AZA, RIVIR_MAZ, RIVEG, RIVCO, RIVTR_ANA |
| 14 | 34.61 | 1.18 | 8 | RIVIT_MED |
| 21 | 44.27 | 1.41 | 8 | RIVIT_MED, RIVIR_AZA, RIVEG, RIVCO, RIVMZ |
| 10 | 97.20 | 1.07 | 8 | RIVIT_MED, RIVMZ |
|  | | | | |
| **SWAMP_DATA** | | | | |
| Chr. | Start (Mb) | Length (Mb) | n. | Breed/population |
| 1 | 11.00 | 1.32 | 7 | SWACN_HUN, SWACN_YAN, SWAIN_JAV, SWAIN_NUT, |
| 2 | 50.8 | 7.60 | 7 | SWACN_FUL, SWACN_GUI, SWACN_YAN, SWACN_YIB, SWAIN_JAV, SWAIN_SUW |
| 2 | 46.26 | 2.76 | 6 | SWACN_ENS, SWACN_FUL, SWACN_GUI, SWACN_HUN, SWAPH, |
| 2 | 49.11 | 71.36 | 6 | SWACN_FUL, SWACN_HUN, SWACN_YIB, SWAPH |
| 1 | 118.12 | 1.16 | 6 | SWACN_ENS, SWACN_YAN, SWAIN_JAV, SWAIN_NUT,  SWATH_THS |
