# Supplementary material for: The distribution of runs of homozygosity in the genome of river and swamp buffaloes reveals a history of adaptation, migration and crossbred events
Source: Genet Sel Evol. 2021 Feb 27;53:20. doi: 10.1186/s12711-021-00616-3 (PMC7912491; doi:10.1186/s12711-021-00616-3)
Supplement: Supplementary file 10 — Additional file 10: Table S4. Chromosomal location of significant SNPs (i.e. with SNPROH values located in the top 1%) in the RIVER_DATA and SWAMP_DATA sets. [file 12711_2021_616_MOESM10_ESM.docx]

|  |  | **RIVER_DATA** |  |  |
| --- | --- | --- | --- | --- |
| Chromosome | n. SNP | position first SNP (Mb) | position last SNP (Mb) | Length (Mb) |
| 1 | 49 | 42.85 | 47.60 | 4.75 |
| 2 | 37 | 49.69 | 54.82 | 5.13 |
| 2 | 4 | 118.00 | 118.11 | 0.11 |
| 3 | 109 | 56.24 | 67.01 | 10.77 |
| 3 | 4 | 76.89 | 77.16 | 0.27 |
| 3 | 3 | 64.25 | 64.39 | 0.14 |
| 4 | 35 | 118.69 | 121.36 | 2.67 |
| 5 | 5 | 26.18 | 26.58 | 0.40 |
| 5 | 38 | 75.95 | 78.63 | 2.68 |
| 6 | 10 | 12.31 | 13.06 | 0.75 |
| 6 | 12 | 45.76 | 46.32 | 0.64 |
| 8 | 16 | 92.71 | 93.84 | 1.13 |
| 9 | 24 | 58.44 | 59.91 | 1.47 |
| 11 | 1 | 18.26 | - | - |
| 13 | 20 | 29.28 | 30.54 | 1.26 |
| 13 | 13 | 52.53 | 53.36 | 0.83 |
| 14 | 4 | 35.57 | 35.75 | 0.18 |
| 16 | 1 | 73.73 | - | - |
| 17 | 4 | 34.18 | 35.44 | 1.26 |
| 18 | 2 | 14.04 | 14.63 | 0.59 |
| 18 | 2 | 50.77 | 50.80 | 0.03 |
| 20 | 11 | 40.69 | 41.42 | 0.73 |
| 21 | 31 | 44.30 | 45.63 | 1.33 |
|  |  |  |  |  |
|  |  |  |  |  |
|  |  | **SWAMP_DATA** |  |  |
| Chromosome | n. SNP | position first SNP (Mb) | position last SNP (Mb) | Length (Mb) |
| 1 | 22 | 11.00 | 12.43 | 1.43 |
| 1 | 21 | 106.60 | 108.99 | 2.39 |
| 1 | 33 | 112.56 | 118.83 | 6.37 |
| 2 | 4 | 44.02 | 44.86 | 0.84 |
| 2 | 23 | 46.84 | 51.43 | 4.59 |
| 2 | 4 | 52.88 | 53.76 | 0.88 |
| 2 | 7 | 55.03 | 56.68 | 1.65 |
| 2 | 7 | 61.62 | 62.41 | 0.79 |
| 2 | 2 | 120.62 | 120.73 | 0.11 |
| 2 | 16 | 127.15 | 128.74 | 1.59 |
| 4 | 11 | 113.92 | 115.01 | 1.09 |
| 4 | 3 | 116.29 | 116.46 | 0.17 |
| 17 | 2 | 50.71 | 50.74 | 0.03 |
| 19 | 11 | 51.63 | 52.29 | 0.66 |
