# Supplementary material for: The distribution of runs of homozygosity in the genome of river and swamp buffaloes reveals a history of adaptation, migration and crossbred events
Source: Genet Sel Evol. 2021 Feb 27;53:20. doi: 10.1186/s12711-021-00616-3 (PMC7912491; doi:10.1186/s12711-021-00616-3)
Supplement: Supplementary file 11 — Additional file 11: Table S5. Genes mapped in the ROH islands highlighted in this study [93–112]. [file 12711_2021_616_MOESM11_ESM.docx]

**Genes annotated in ROH islands detected on ALL_DATA set**

| **Position (Mb)** | **Gene name** | **Function** | **Associated traits** | **Reference** |
| --- | --- | --- | --- | --- |
| Chr1:11.12-11.30 | *ADAM Metallopeptidase Domain 32* (*ADAM32)* | Cell surface protein | Mouse sperm development | [36] |
| Chr1:11.30-11.39 | *ADAM Metallopeptidase Domain 9* (*ADAM9*) | Cell surface protein | Cattle follicle development | [37] |
| Chr1:11.42-11.50 | *Pleckstrin Homology Domain Containing A2* (*PLEKHA2*) | Cell signaling pathways | Cattle follicle development.  Mouse embryonic development, | [37,93] |
| Chr1:11.92-11.98 | *Fibroblast Growth Factor Receptor 1* (*FGFR1*) | Regulation of ovarian angiogenesis | Corpus luteum activity in cattle,  Number of piglets born alive | [38,39] |
| Chr1:11.70-11.72 | *Transfer RNA Glycine (Anticodon CCC)* *(*TRNAG*)* | RNA processing | Growth traits in Buffalo and Bos indicus | [94,95] |
| Chr1:112.45-112.61 | *Adenylate Cyclase 5* (*ADCY2)* | Transmembrane protein | Cow fertility | [96] |
| Chr1:113.22-113.92 | *Kalirin RhoGEF Kinase* (*KALRN*) | Cell signaling pathways | Cow fertility  Response to Mycobatterium avium spp. Paratuberculosis in cattle | [96-98] |
| Chr1:113.92-113.98 | *Uridine Monophosphate Synthetase* (*UMPS*) | Pyrimidine synthesis | Feed efficiency in cattle | [41] |
| Chr1:113.99-114.12 | *Integrin Subunit Beta 5* (*ITGB5*) | Cell surface receptor | Resistance to Escherichia coli F4ac adhesion to intestinal mucosa in pigs | [98,100,101] |
| Chr1:114.13-114.15 | *Mucin 13, Cell Surface Associated* (*MUC13*) | Cell surface glycoprotein | Resistance to Escherichia coli F4ac adhesion to intestinal mucosa in pigs | [98,100,101] |
| Chr1:114.22-114.30 | *Heart Development Protein With EGF Like Domains 1* (*HEG1*) | Cell signaling pathways receptor | Resistance to Escherichia coli F4ac adhesion to intestinal mucosa in pigs  Mycobatterium avium spp. Paratuberculosis in cattle (LRCH3) | [98,100,101] |
| Chr1:115.03-115.13 | *Leucine Rich Repeats And Calponin Homology Domain Containing 3* (*LRCH3*) | Regulation of the actin cytoskeleton | Resistance to Escherichia coli F4ac adhesion to intestinal mucosa in pigs  Mycobatterium avium spp. Paratuberculosis in cattle (LRCH3) | [98,100,101] |
| Chr1:114.97-115.03 | *IQ Motif Containing G* gene (*IQCG*) | Ciliary motility | Semen quality traits in bulls and pigs | [40,99] |
| Chr2:47.27-47.35 | *GDNF Family Receptor Alpha Like* (*GFRAL*) | Receptor of growth differentiation factors | Body energy balance in mice | [102] |
| Chr2:48.54-49.16 | *Dystonin* (*DST)* | Adhesion junction plaque protein | Spermatozoa motility, daughter still birth in cattle | [42,43] |
| Chr2:49.33-49.36 | *Member RAS Oncogene Family* (*RAB23*) | Regulation of intracellular membrane trafficking | Oestrus display in pigs | [44] |
| Chr2:51.36-52.23 | *KH RNA Binding Domain Containing, Signal Transduction Associated 2* (*KHDRBS2*) locus | Regulation of RNA alternative splicing | Reproduction in cattle, goat, and pig | [45-47] |
| Chr2:52.90-52.97 | *Lengsin, Lens Protein with Glutamine Synthetase Domain* (*LGSN*) | Pseudo Glutamine synthetase | Metabolism of the Glutamate in the eye retina | [49] |
| Chr2:52.97-53.35 | *Type II oculocutaneous albinism* (*OCA2*) | Tyrosine transportation | Skin pigmentation in humans  Eye disease in horse | [50]  [51] |
| Chr2:53.31-53.56 | *HECT and RLD Domain Containing E3 Ubiquitin Protein Ligase 2* (*HERC2*) | Pigmentation metabolism | Iris heterochromia in humans | [52] |
| Chr2:53.68.53.77 | *Cytoplasmic FMR1 Interacting Protein 1* (*CYFIP1*) | Cytoskeletal dynamics | Nematode resistance in sheep | [104] |
| Chr2:53.85-53.91 | *Tubulin Gamma Complex Associated Protein 5* (*TUBGCP5*) | Microtubule nucleation at the centrosome | Fertility in humans | [48] |
| Chr2:53.94-53.96 | *Protein Tyrosine Phosphatase Non-Receptor Type 18* (*PTPN18*) | Signal protein | Feeding behavior in pigs | [54] |
| Chr2:54.08-54.09 | *APC Membrane Recruitment Protein 3* (*AMER3*) | Signal protein | Embryogenesis | [55] |
| Chr2:54.11-54.48 | *Rho Guanine Nucleotide Exchange Factor 4* (*ARHGEF4*) | Signal protein | Milk production in cattle | [56] |
| Chr2:54.5-54.56 | *Pleckstrin Homology Domain Containing B2* (*PLEKHB2*), | Protein trafficking | Residual feed intake in cattle  Follicle size in cattle  Mastitis resistance in sheep | [37,57,103] |
| Chr2:57.32-57.37 | *LIM Zinc Finger Domain Containing 2* (*LIMS2*) | Adhesion protein | Respiration rate in pigs | [105] |

**Genes annotated in ROH islands detected on RIVER_DATA set**

| **Position (Mb)** | **Gene name** | **Function** | **Associated traits** | **Reference** |
| --- | --- | --- | --- | --- |
| Chr1:40.66-42.63 | *CUB And Sushi Multiple Domains 1* (*CSMD1*) | Regulator protein | Development of central nervous system | [112] |
| Chr1:43.36-43.43 | *Myomesin 2* (*MYOM2*) | Cytoskeleton protein | Muscle contraction | [60] |
| Chr1:43.45-43.46 | *Kelch repeat and BTB domain-containing protein 11* (*KBTBD11*) | Signal protein | Osteoclast differentiation | [109] |
| Chr1:43.47-43.53 | *Rho guanine nucleotide exchange factor 10* (*ARHGEF10*) | Metabolism of fatty acid desaturases | Hairless phenotype in pigs | [62,63,110,111] |
| Chr1:43.62-43.68 | *Transmembrane ER and ERGIC Protein* (*CLN8*) | Vacuolar transport | Hairless phenotype in pigs | [62,63,110,111] |
| Chr1:43.69-44.30 | *Disk Large Scaffolding Associated Protein 2* (*DLGAP2*) | Transmission of neuronal signals | Hairless phenotype in pigs | [62,63,110,111] |
| Chr1:46.72-46.75 | *Interferon Gamma Receptor 2* (*IFNGR2*) | Receptor protein | Polledness in cattle and yak | [64-66] |
| Chr1:46.81-46.84 | *Interferon Alpha And Beta Receptor Subunit 1* (*IFNAR1*) | Receptor protein | Polledness in cattle and yak | [64-66] |
| Chr1:47.45-47.54 | *Synaptojanin 1* (*SYNJ1*) | Phosphatase | Polledness in cattle and yak | [64-66] |
| Chr3:57.46-57.47 | *Transmembrane Protein 100* (*TMEM100*) | Signal protein | Height in humans | [106] |
| Chr3:57.85-57.89 | *HLF transcription factor* (*HLF*) | Ion channel protein | Adaptation to specific environments | [59] |
| Chr3:57.94-58.00 | *Monocyte to macrophage differentiation associated* (*MMD)* | Transcription factor | Adaptation to specific environments | [59] |
| Chr3:58.13-58.41 | *Syntax Binding Protein 4* (*STXBP4*) | Translocation of transport vescicles | Adaptation to specific environments | [59] |
| Chr3:63.42-63.50 | *Annexin a10* (*ANXA10*) | Cellular growth regulation | Embryonic mortality in cattle | [107] |
| Chr3:64.08-64.23 | *SH3 domain containing ring finger 1* (*SH3RF1*) | Protein ligase | Parasite resistance in sheep | [104] |
| Chr3:64.85-64.87 | *Aminoadipate Aminotransferase* (*AADAT*) | Transaminase | Body weight in cattle | [108] |
| Chr4:118.80.118.93 | *Leucine Rich Repeat Containing G Protein-Coupled Receptor 5* (*LGR5*) | Transmembrane receptor | Supernumerary teats in cattle | [69] |
| Chr8:93.43-93.54 | *Ubiquitin Conjugating Enzyme E2 H (UBE2H)* | Cellular protein degradation | Cattle feed efficiency | [68] |
| Chr9: 58.96-58.97 | *Protocadherin Beta 7* (*PCDHB7*) | Plasma membrane protein | Milk protein composition in cattle | [69] |
| Chr9: 58.47-58.48 | *CD14 molecule* (CD14) | Surface antigen | Immune response to mastitis | [70] |
| Chr18:14.15-14.17 | *Cyclin Dependent Kinase 10* (*CDK10*) | Phosphorylation protein | Immune response | [71] |
